# Influence of Nicotine from Diverse Delivery Tools on the Autonomic Nervous and Hormonal Systems

**DOI:** 10.3390/biomedicines10010121

**Published:** 2022-01-06

**Authors:** Valerii A. Menshov, Aleksei V. Trofimov, Alla V. Zagurskaya, Nadezda G. Berdnikova, Olga I. Yablonskaya, Anna G. Platonova

**Affiliations:** 1 Emanuel Institute of Biochemical Physics, Russian Academy of Sciences, 119334 Moscow, Russia; berdnad@mail.ru (N.G.B.); olga.yablonsky@gmail.com (O.I.Y.); 2 Moscow Institute of Physics and Technology (National Research University), 141701 Dolgoprudny, Russia; 3 Medical Center AVC & DNKom Laboratory, 119334 Moscow, Russia; alla.viktorova.2014v@mail.ru; 4 Department of Clinical Pharmacology, I.M. Sechenov First Moscow State Medical University, 119991 Moscow, Russia; 5 MedBazis LLC, 190013 Saint Petersburg, Russia; platonova@medbazis.com

**Keywords:** nicotine, oral nicotine packs, heart rate variability, neurohormones, cortisol, nicotine-free herbal cigarettes, autonomous nervous system, carbon monoxide

## Abstract

Background: Through measurements of the heart rate variability (HRV) accompanied by the pertinent biomarker assays, the effects of nicotine and byproducts derived from alternative nicotine delivery systems (ANDS) on the autonomic nervous system (ANS) and hormonal system have been investigated. Methods: HRV was studied in a group of volunteers (17 people), involving non-smokers, i.e., who never smoked before (11), ex-smokers (4) and active smokers (2). ANDS and smoking simulators, including regular, nicotine-free and electronic cigarettes; tobacco heating systems; chewing gums and nicotine packs of oral fixation (nic-packs), were used. Blood pressure, levels of stress hormones in saliva and catecholamines in the blood were also monitored. Results: HRV analysis showed relatively small changes in HRV and in the other studied parameters with the systemic use of nic-packs with low and moderate nicotine contents (up to 6 mg) compared to other ANDS. Conclusions: The HRV method is proven to be a promising technique for evaluation of the risks associated with smoking, dual use of various ANDS and studying the biomedical aspects of smoking cessation. Nic-packs are shown to be leaders in biological safety among the studied ANDS. A sharp surge in the activity of the sympathetic division of the ANS within the first minutes of the use of nicotine packs implies that nicotine begins to act already at very low doses (before entering the blood physically in any significant amount) through fast signal transmission to the brain from the nicotinic and taste buds located in the mouth area.

## 1. Introduction

The importance and topicality of the study reported herein stem from the impetuous spreading over the consumer market the new alternative to traditional tobacco products, nicotine delivery tools, which science and practical medicine do not yet keep up with in terms of studying and understanding the biological consequences of using the novel nicotine delivery systems. Clearly, there exists a call and need for a rapid and efficient methodology that can keep up with the rapidly changing modes of nicotine delivery. Thus, the objective of this study consisted of searching for an appropriate methodology and of its experimental approbation through studying the pertinent biological impacts of different means of the nicotine delivery. In this context, the following two circumstances are particularly noteworthy. The first circumstance refers to a crucial and multifaceted role of the autonomic nervous system (ANS) in functioning diverse physiological systems in living organisms, while the second one pertains to the fact that nicotinic cholinergic receptors (nAChRs) are widespread in the body and thereby account for the multifarious biological effects of nicotine upon its consumption [1,2,3]. Hence, the question arises of how different the effects of nicotine from various systems of its delivery will be on the functioning of the ANS, which controls the key physiological processes. Can the observed differences in the effects of nicotine on the ANS be harnessed for comparing the risks of using various nicotine products? The need to resolve these issues scientifically substantiates the purpose and rationale of this study.

The heart rate variability (HRV) indices provide established diagnostic and prognostic markers of the ANS functional state [4,5]. Thus, the strategy behind our experimental approach refers to measuring and analyzing the HRV characteristics while consuming various types of nicotine products. In view of the linkage between the activity of the ANS and hormonal system [6], we also performed an analysis of the key hormones as a function of consuming diverse nicotine products.

Our main working hypotheses pertain to the following contentions: (i) the HRV-based methodology should enable a rapid assessment of the differences between the biological impacts of nicotine derived from different delivery systems, (ii) smokeless nicotine-contained products are expected to exhibit reduced and moderate effects on ANS and hormonal system compared to the influence of traditional cigarettes and (iii) taste buds located in the human mouth area may be sensitive to very low doses of nicotine, causing a response from the ANS.

The present study was based on contemporary knowledge, and an overview is given below.

It is well-known that, with few exceptions, almost all mammalian internal organs are under control by the ANS. Information about its state is permanently transmitted to the central nervous system (CNS) by sensory afferent fibers. The CNS integrates sensory signals and sends neural commands back to the organ through the autonomous ganglia. The cranial cervical ganglion is the most important integrative and coordinating center of autonomous regulation. Through nAChRs of the cranial cervical ganglion, the general vascular tone and hemodynamic parameters are controlled, along with pineal gland activity and the regulation of many other vital visceral functions [1]. In the event of a malfunction of any organ, the tasks of the ANS include not only providing signal information to the brain but also eliminating the consequences of disorders by mobilizing the body’s resources, maintaining homeostasis and increasing its adaptive capabilities.

There is no doubt that the metastatic process in carcinogenesis is a key cause of cancer death, including smoking-related lung cancer. Notably, nicotine in this process plays only an indirect role. Until now, there has been no clear understanding of how various components of cigarette smoke modulate the activity of the ANS and whether these changes are the cause or consequence of neoplasms.

Tumor cells express different neurotransmitter receptors and react with different neuromodulators secreted by the ANS from the brain, peripheral plexuses, ganglia and adrenal medulla [2]. It is becoming more and more obvious that the growth of a malignant tumor is largely related to signals from the nervous system [7,8,9,10].

Stimulation of the vagus nerve, which activates the parasympathetic nervous system, inhibits the release of sympathetic neurotransmitters [11]. Gidron and coauthors showed that stimulation of the vagus nerve controls and reduces tumor progression [12]. Mid-stage tumors are often accompanied by damage to autonomic nerves, and a decrease in HRV is a typical symptom in patients with advanced tumors [13]. Clinical studies have shown that HRV can be used to predict the severity and outcome in many types of cancer, including liver [13], breast [14] and colon [15] cancers.

Not so long ago, a detailed report on long-term studies of the relationship of HRV with the etiology of gastric cancer was published [16]. For 5 years, various HRV parameters were measured by ECG in 383 patients diagnosed with gastric cancer, including the standard deviation of the normal RR intervals (SDNN) and the root mean square value of the differences between successive RR intervals (RMSSD). HRV was found to correlate with the tumor size, tumor infiltration, lymph node metastasis and distant metastasis; however, no correlation was found with tumor localization and severity of the metastasis.

HRV was a predictor of mortality not only in cancer patients but also, for example, in chronic kidney disease [17]. Using the same HRV markers (SDNN and RMSSD), Drawz et al. came to the conclusion that the RMSSD indicator has the greatest information content and diagnostic value.

Although mechanistic studies have shown that smoking is associated with the chronic stimulation of the sympathetic division of the ANS and, as a result, with a decrease in HRV, population studies often provide conflicting results. The controversy sometimes reaches the point that the actual results of the study (initial data) do not coincide with the conclusions of the authors [18]. The reason for this disagreement lies primarily in the subtle opposition of the two systems—namely, the sympathetic and parasympathetic divisions of the ANS, where nicotine is the common playmaker. Smoking (cigarette smoke and nicotine), being an agonist of both choline and adrenergic receptors, in the short term (seconds and minutes after smoking), stimulates predominantly sympathetic activity, while, over a longer time interval (tens of minutes and hours), there is an increasing trend in the activity of the parasympathetic department. The situation is aggravated by the fact that the mechanisms of the influence on HRV in nicotine and other components of smoke can differ significantly. However, special studies devoted to this issue, apparently, have never been carried out, despite the fact that a large number of works have referred to studying the effect of air pollution on the activity of the ANS [19,20,21,22,23,24,25,26,27] (an overview of these studies is given in the Supplementary Materials).

In this aspect, the study of the properties of nicotine-free smoke is extremely important for predicting the long-term risks associated with the use of ANDS by both former and current smokers. Combining regular cigarette smoking with ANDS can have the most negative impact on the health of smokers.

As shown by the results of recent clinical studies [28], the propensity towards various diseases in smokers who use both traditional cigarettes and ANDS increases significantly compared to those smokers who use only traditional cigarettes or only ANDS. This paradox has not yet found a clear explanation, except that the level of nicotine increases in the body of dual users. However, not all of the disease risks in dual users can be attributed to a simple increase in nicotine intake or ANDS characteristics. After the start of the use of ANDS in the smoker’s body, the ratio between the concentration of nicotine (cotinine) and other components of smoke, and primarily such basic ones as carbon and nitrogen monoxides, tar, etc., changes dramatically.

Apparently, the adaptation of the body of a smoker using conventional cigarettes and ANDS to the altered balance of the main macro-components of the smoke and aerosol does not occur quickly and without a trace. As a result, dual users are more prone to cardiovascular and endocrine diseases, as well as dysfunctions of the respiratory and immune systems. This should be particularly true for smokers with many years of experience who gradually develop a tolerance, adaptation and, in some way, synchronization of the body’s response to nicotine and, for example, CO in a certain proportion. In the case of a sharp cancellation of the intake of CO (as can occur with a complete or partial cessation of smoking in favor of ANDS) or a sharp increase in the intake of nicotine (when any ANDS is added to the usual amount of cigarettes smoked per day), an imbalance of the ANS may be observed.

The appearance on the market of many new systems for the delivery of nicotine, alternative to conventional cigarettes, on the one hand, has contributed to the cessation of smoking and the overall reduction of the risk of diseases, and on the other hand, it has further complicated the situation and created a problem for the prompt and personalized diagnosis of the consequences of their use.

Summarizing all the aforesaid, in the study disclosed herein, we have examined in detail the activity of the ANS with the help of a HRV analysis applied to volunteers using various nicotine delivery systems and online smoking simulators. Special emphasis in the work was placed on the novel product on the market—namely, nicotine tobacco-free packs, which, for certain reasons, have received an ambiguous resonance in the public opinion.

## 2. Materials and Methods

### 2.1. Volunteers

The study involved a group of volunteers (17 people) of non-smokers (11), ex-smokers (4) and active smokers (2). Informed consent was obtained from all of them for participation in the study and processing of their personal and biomedical data. All volunteers were between the ages of 33 and 63 years, had a normal body mass index, no serious chronic diseases, no alcohol abuse and no medication for at least 2 weeks prior to the study.

### 2.2. ECG Measurements and HRV Analysis

To obtain an ECG signal, analyze them and acquire RR intervals, we used the VedaPulse Pro hardware and software complex (manufactured by Biokvant LLC, Russia), FDA-certified equipment [29]. The ECG was recorded in a sitting position.

The acquisition of HRV data and their analysis were done according to the standard procedures [4,5] as follows. Heart beats were automatically registered and then reviewed by the operator who detected mislabeled beats or artifacts. Normal-to-normal (NN) beat intervals were the major characteristics for the HRV analysis. Other parameters that were registered and computed were the standard deviation of NN intervals (SDNN), the square root of the mean of the squared differences between adjacent NN intervals (RMSSD), low-frequency power (LF) (0.04–0.15 Hz), high-frequency power (HF) (0.15–0.4 Hz) and the LF/HF ratio. Individuals with atrial fibrillation, atrial bigeminy and trigeminy, pacemakers, irregular rhythm, irregular sinus rhythm, frequent ventricular ectopic activity, ventricular bigeminy or multifocal atrial tachycardia were excluded from the study.

SDNN is predominantly (but not exclusively) an indicator of sympathetic activity (at long intervals of ECG recordings), whereas RMSSD and pNN50 (percentage of RR intervals longer than 50 milliseconds) are associated with parasympathetic activity. The Kaplan respiratory modulation index was calculated as IDM = (0.5 * RMSSD/RRNN) * 100%.

The HF value is an indicator of parasympathetic activity, while LF correlates with sympathetic and parasympathetic activity, and the VLF component of the spectrum is often associated with the activity of hormonal systems. The total spectral power (TP) is a broad measure of the total vegetative activity. Modulation of the parasympathetic activity (defined as vagal heart tone) is also associated with the regulation of emotional reactivity, psychological flexibility and social activity, as well as a decrease in prefrontal cortical activity [30].

### 2.3. Biochemical Analyses

Analysis of the hormones (cortisol and estradiol in saliva and, additionally, adrenaline, noradrenaline, dopamine, serotonin, adrenocorticotropic hormone and somatotropic hormone in the blood) was carried out on the basis of the medical diagnostic center AVC (Moscow) by HPLC-MS. Analysis of cotinine and nicotine in the saliva was determined by gas chromatography and mass-spectroscopy (GC-MS). Statistical processing and graphical presentation of data were performed using Statgraphics version XVII and SigmaPlot version 12.

The level of carboxyhemoglobin (%HbCO) in the blood of the volunteers was determined on a Smokerlyzer piCO device (Bedfont Scientific, Ltd., UK), according to the instructions. When testing nicotine-free cigarettes with non-smoking volunteers, the subjects smoked a cigarette, taking 9 deep puffs every 30 s.

The predictive and clinical significance determining the concentration of nicotine and cotinine in the saliva was assessed in accordance with the studies of Teneggi et al. [31], who evaluated the predictive performances of saliva. They showed that saliva was a useful alternative to plasma for the routine monitoring of nicotine. According to the reported data, the deviation with the predicted plasma concentrations was of 0.47 ng/mL with a 95% confidence interval of (−0.57 to 1.52) and a precision of 5.68 ng/mL. The authors concluded that an analysis of saliva is potentially an easy and noninvasive promising way to evaluate the nicotine concentration. Saliva sampling does not cause discomfort compared to venous punctures in most individuals. Psychological studies of the subjective moods of donors have shown that blood collection causes pain and anxiety. Therefore, saliva can be considered as the ideal test material for clinical studies that evaluate the effect of drugs for nicotine deprivation symptoms treatment.

### 2.4. Microbiological Analyses

The qualitative and quantitative compositions of the microflora of the small intestine were determined by the content of microbial markers using GC-MS [32]. The method of mass spectrometry of microbial markers (MSMM) is based on the highly accurate determination of specific marker molecules that make up the cellular lipids of microorganisms. They can be detected in the blood and other clinical materials by a highly sensitive and selective method of GC-MS against the dominant background of the lipid substances of the material itself. The method allows one to simultaneously measure the concentration of more than a hundred microbial markers directly in the analyzed materials: blood, urine, biopsies and other biological fluids and tissues, as well as in nonbiological samples, bypassing the stage of pre-inoculation on nutrient media or the use of test biochemical materials [33].

### 2.5. Nicotine Delivery Tools

Tobacco heating systems (IQOS and glo); electronic cigarettes (pod systems) JUUL, Compact Logic and myBlu (with salt and free (free-base) nicotine), tank-type nicotine-free electronic cigarettes (open system) Vaporesso with a ceramic vaporizer filled with 100% propylene glycol; Nicorette chewing gum (4 mg nicotine) and tobacco-free nicotine packs with nicotine contents of 4 mg and 5.6 mg were used as the ANDS.

The details on testing nicotine-free cigarettes, ECG recordings and sampling the biological fluids, as well as the peculiarities of processing the experimental data [34], are disclosed in the Supplementary Materials).

## 3. Results

### 3.1. The Role of Smoke Components in Modulation of ANS Activity

**Figure 1:**
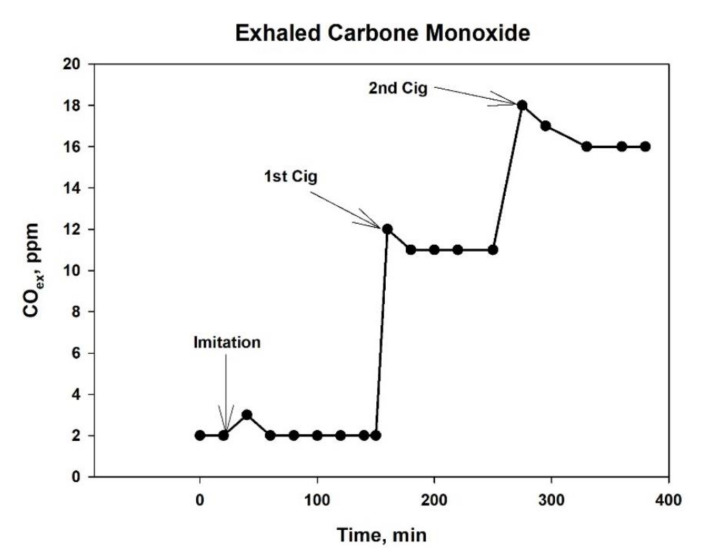
CO dynamics in the exhalation of a volunteer after smoking two nicotine-free cigarettes with an interval of 90 min.

To study the effect of smoke on the ANS, we took advantage of Nirdosh tobacco-free cigarettes, which have long been used in Oriental meditation practice and are manufactured on an herbal basis with a cellulose acetate filter. With the help of such cigarettes, it is relatively safe to study the effect of smoke on the bodies of non-smokers under conditions as close as possible to smoking a regular cigarette, including all the attributes inherent in smoking. The tar yield in the smoke of such cigarettes reaches 22 mg without the use of special tar capacitors in the form of filter mouthpieces and less than 6 mg with the use of such devices, which is more consistent with the tar delivery in modern cigarettes of a moderate strength. The preliminary tests showed that removing a significant portion of the tar from cigarette smoke did not affect the CO concentration in the smoke. The typical profile of the CO dynamics in the exhalation of a volunteer after smoking two cigarettes is exemplary shown in Figure 1.

In order to exclude possible psychosomatic reactions reflexively arising in a smoker in response to imitation smoking, we recruited volunteers as test subjects without a history of smoking and nicotine consumption with an exhaled CO level of no more than 3 ppm.

As noted earlier in the Materials and Methods section, the measurement of the heart rate (HR) and HRV parameters directly during smoking a cigarette or simulating smoking can be associated with a poorly controlled influence of a number of factors and lead to artifacts that worsen the power of a statistical analysis. For this reason, we were very cautious and critical of including each RR measurement from the cigarette smoking period into the analytical procedures. On the other hand, the process of recording cardiograms at intervals of 5–8 min (depending on the heart rate) with breaks of 15–20 min (to restore the balance of physical activity) does not allow collecting enough data in a relatively short period of time (5–6 h) to obtain statistically reliable results. Therefore, to assess the statistical reliability, we made a separate analysis of the reliability of the differences in the group mean values of the RMSSD indicator with the inclusion and exclusion of the data obtained directly during the smoking session.

**Figure 2:**
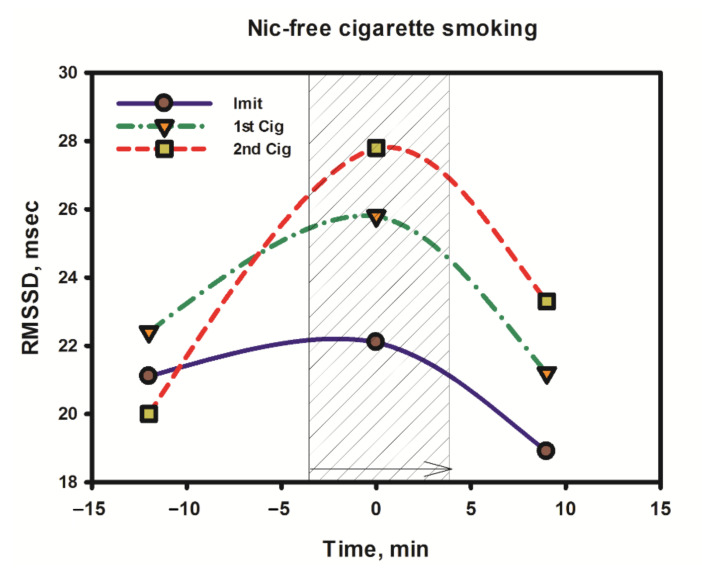
Increase in parasympathetic activity in a volunteer when smoking two nicotine-free cigarettes (central points on the graph) with an interval of 90 min but not imitating smoking (Imit) in comparison with the pre-smoking and post-smoking stages.

As follows from Figure 2, the minimum increase (or lack thereof) in the RMSSD value and the putative parasympathetic activity was observed at the stage of simulating smoking, when the subject, instead of smoke, inhaled air through a cigarette, holding it in a hand and making characteristic puffs and breathing movements similar to those the smoker makes when smoking a regular cigarette. A slightly higher increase in parasympathetic activity was observed during smoking of the first cigarette. Then, with a repeated smoking session (with an interval of 1.5 h after the first cigarette), the increase in parasympathetic activity was the most pronounced. It should be emphasized here that the parasympathetic stimulation by smoke (smoking) began to manifest itself literally from the first deep inhalation when smoking a cigarette, and it was clearly seen on the infographics during online testing using the VedaPulse Pro hardware and software complex (Release 10 December 2020).

**Figure 3:**
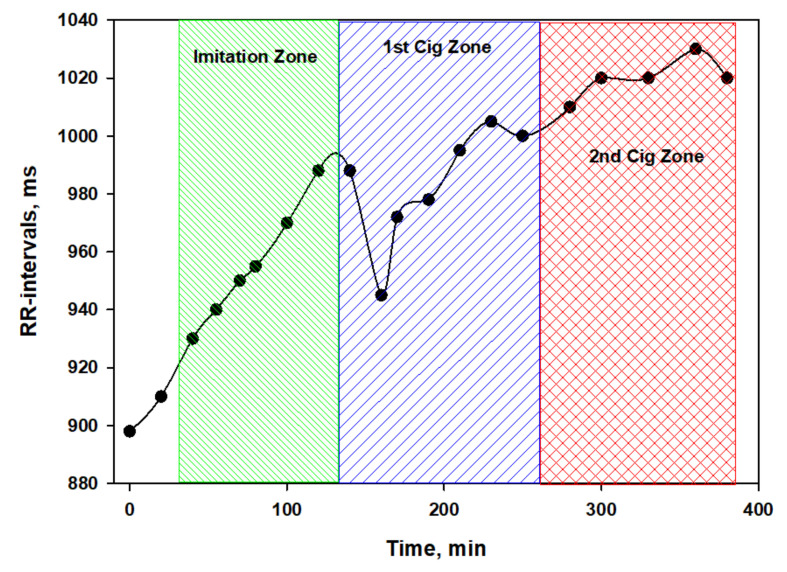
Dynamics of the RR intervals of a volunteer at different stages of testing a nicotine-free cigarette. To accurately determine the correspondence of the time coordinates of each stage of the experiment in the figure, the test protocol should be used. Green color—imitation of smoking, blue color—1st cigarette zone, red color—2nd cigarette zone. (Table S1, Supplementary Materials).

Besides, considering the duration of the experiment (more than 6 h for each subject), probably HRV indicators need to be corrected, taking into account the HR trend. The validity of this requirement is clearly seen in Figure 3, which shows the dynamics of the RR intervals of a volunteer on the day of testing a nicotine-free cigarette.

**Figure 4:**
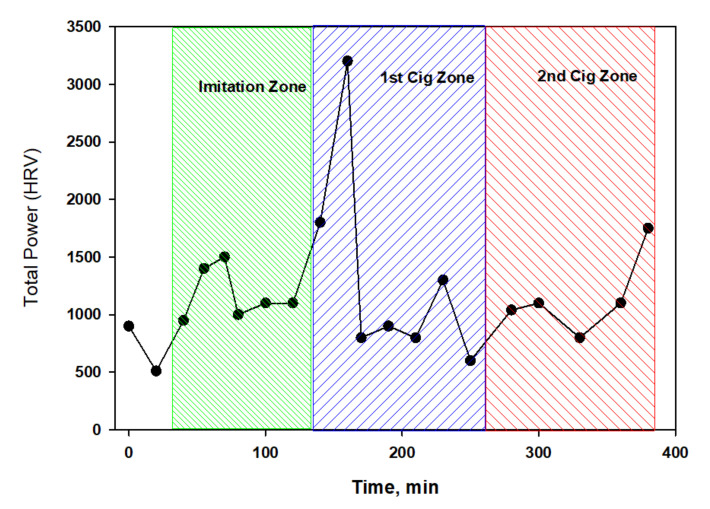
The dynamics of the total power spectrum (TP) during the testing of nicotine-free cigarettes.

**Figure 5:**
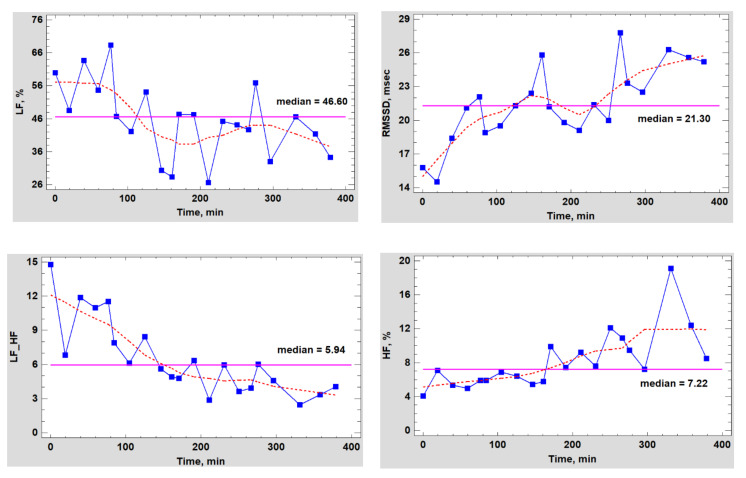
Dynamics of HRV indicators in a volunteer during the testing period for nicotine-free cigarettes.

Interestingly, the total power (TP) spectrum fluctuated slightly around the average values (Figure 4) against the background of significant changes in the heart rate. This characterizes nicotine-free smoke as an inactive modulator of ANS. However, this conclusion can be easily refuted if we consider in detail the dynamics of different parts of the spectrum (Figure 5).

As can be seen from the data in Figure 5, the HF index almost did not change in the control experiment (0–60 min) or within an hour after the completion of the simulation of smoking (80–140 min), but immediately after smoking the first cigarette, the trend began to change rapidly, and the parasympathetic activity began to grow rapidly and in waves. However, the dynamics of the LF component had a completely different characteristic, and it cannot be said that it was the opposite of the one mentioned above. The LF component decreased to almost its minimum by the end of the experiment with simulated smoking, and later, it only fluctuated around this value. Apparently, nicotine-free smoke caused neither psycho-emotional or physiological stress in the subject, which was also confirmed by the absence of blood pressure surges. Conversely, the fact that there was a noticeable shift in the balance of the ANS towards an increase in vagal activity was also evidenced by the dynamics of the RMSSD indicator, a detailed statistical analysis of which was carried out both with and without taking into account the changing heart rate trend during the tests.

In the Supplementary Materials, we report the detailed statistical analysis on the comparison of the RMSSD by the stages of cigarette testing. The pertinent data include the results of performing the multiple range tests for the RMSSD indicator with and without taking into account the smoking session (Table S2), estimated difference between each pair of means (Table S3), multiple range tests (95% Games-Howell, Table S4) and the results of the two-way analysis of variance MANOVA of testing a series of the two nicotine-free cigarettes in non-smokers with different ANS status (Table S5). Besides, the RMSSD dynamics in the process of testing the nicotine-free cigarettes depending on the ANS type is depicted in Figure S1, while the RMSSD dynamics relative to sham smoking is displayed in Figure S2. 

The composition of cigarette smoke, both in gas and in solid phases, contains numerous products that can change the heart rate both in the direction of its acceleration and in the direction of slowing down. Among them, the most important are carbon monoxides (CO) and nitrogen (NO), particles with a diameter of less than 2.5 microns (PM_2.5_) and some tar components. For example, CO can have a direct and indirect effect on HRV, causing hypoxia in the organs. As follows from the data presented above (Figure 1), just one smoked nicotine-free cigarette with a filter increases the level of carboxyhemoglobin in the blood 2.5 times, and repeated smoking sessions with an interval of 1.5 h increases it six times. Moderate hypoxia stimulates heme oxygenase and the production of bilirubin, which increases HRV through the mechanism of suppression of sympathetic ganglionic signaling through the inhibition of postganglionic receptors nAChRs [35]. In addition, numerous case studies have described cardiac abnormalities in the form of repolarization, arrhythmias and prolongation of the QT interval even after mild carbon monoxide poisoning [36].

It has been shown that proarrhythmic effects of CO arise as a result of the activation of NO synthase, which leads to NO-mediated nitrosylation of the sodium channel protein NaV1.5 and the induction of the late Na^+^ current [37]. Under chronic exposure, CO contributes to the pathological phenotype of cardiomyocytes in the absence of underlying cardiomyopathy. The less severe phenotype in vivo suggests the participation of compensatory mechanisms; according to which, the tendency to arrhythmia may arise due to intracellular Ca^2+^ overload [38].

Taken together, on the basis of the mechanisms considered, it cannot be unequivocally stated that, by increasing the HRV, CO has an exclusively pathological effect on the ANS and the body as a whole, although, in combination with other negative factors, the picture may be just that. Many authoritative researchers believe that CO is the most paradoxical molecule, not only from exogenous sources but also at the endogenous level. While short-term exposure to low doses of CO can be beneficial and cardioprotective, protecting cardiomyocytes from oxidative stress and improving the blood supply to the heart by dilating the coronary vessels, long-term exposure to high doses leads to pathological changes, mainly due to changes in the redox status, ionic homeostasis, intracellular Ca^2+^ pool and sympato-vagal imbalance [39,40]. In other words, a well-known principle works in relation to CO when too much good sooner or later turns into something harmful.

In addition to CO, there are other products in the composition of smoke that can significantly affect the HRV even at many times lower doses than those recorded in cigarette smoke. First of all, these are solid components of smoke that form the basis of the resin. Among them, the main role is played by monoamine oxidase inhibitors found both in tobacco and in its pyrolysis products [41]. It was found that the inhibition of monoamine oxidases can contribute to an increase in heart rate variability [42], possibly by increasing the bioavailability of neurohormones from the class of catecholamines.

**Figure 6:**
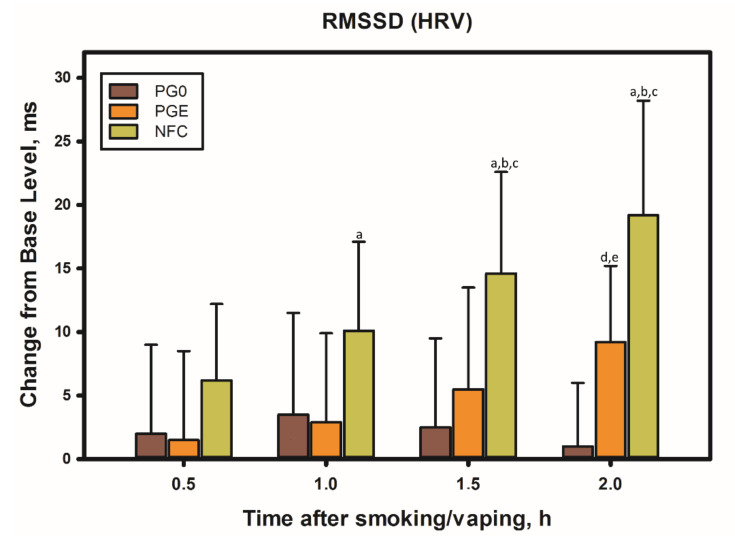
Influence of the smoke extract of the nicotine-free cigarette tar, free of the gas-phase components, on HRV in four non-smoking volunteers. PG0 refers to 25 puffs of the electronic cigarette with propylene glycol (control), and PGE pertains to 25 puffs of the electronic cigarette with the smoke extract, while NFC denotes 10 puffs of a Nirdosh nicotine-free cigarette. Designations of statistically significant differences (*p* < 0.05): a—relative to the baseline in the experiment with smoking (t = 0 h), b—in the experiment with smoking relative to the electronic cigarette with propylene glycol (PG0), c—in the experiment with smoking relative to the electronic cigarette with the cigarette-smoke tar extract (PGE), d—in the experiment with the electronic cigarette with the cigarette-smoke tar extract relative to the baseline (t = 0 h), e—in the experiment with the electronic cigarette with the tar extract (PGE) relative to the electronic cigarette with propylene glycol (PG0).

Using tobacco-free and nicotine-free analogs of cigarettes, we tested the hypothesis about the effect of solid-phase components of smoke (tar) on HRV. For this, a propylene glycol extract of the tar obtained by smoking Nirdosh nicotine-free cigarettes using a Borgwaldt A14 smoking machine was prepared. Then, the extract, containing 60 mg of the tar in 10 mL of solvent, was immediately used as an e-cigarette filler and tested on volunteers in doses comparable to those they received when smoking a regular cigarette (Figure 6).

As it turned out, during the first hour after the vaping session with the tar extract, the HRV remained practically unchanged. However, after 1.5 h, the effect reached 20% of the value obtained after smoking a cigarette (PGE-PG0)/NFC), and after another half an hour, it was already 50%. Apparently, the difference in time of action of different fractions of smoke is explained by different rates of assimilation (metabolism) of the solid and gas components of the smoke. Products in the gas phase almost instantly enter the bloodstream and are distributed to organs and tissues, including the brain and central nervous system, while solid-phase tar components, such as polyphenols, etc., enter the bloodstream mainly through the gastrointestinal tract and oral mucosa, often being exposed to pathways of biotransformation. The main tobacco polyphenols are rutin and chlorogenic acid, which together account for up to 3% of the weight of tobacco, while the highest content of phenolic substances in the smoke does not exceed 300 μg/cigarette (which is two orders of magnitude lower than in the cigarette tobacco), and the main polyphenols are hydroquinone and pyrocatechol [43].

Due to the close connection between the CNS and the microbiota present in the gastrointestinal tract, the gut–brain axis plays an important role in the stress response and is admittedly part of the neuroendocrine system [44]. Metabolites of dietary polyphenols produced by the gut microbiota are well-absorbed in the gut and are able to circulate in the plasma for a longer time. In contrast, low molecular weight polyphenols can be directly adsorbed in the small intestine [45] and reach the brain much faster unchanged. Stimulation of the parasympathetic division of the ANS can also occur in the intestine itself, bypassing the central nervous system [46].

As shown by the experiments of Duarte et al. [47], within an hour after consuming 10 g of dark chocolate containing only 5.2 mg of polyphenols, the average RMSSD value in a group of 21 volunteers increased from 34 ms to 55 ms, while, in the group that ate polyphenol-free white chocolate, this indicator decreased by 8 ms [47]. This experience clearly shows how polyphenols, even in very small doses, can affect the ANS through the gastrointestinal tract, but the exact mechanism of influence has not yet been established.

Our tests have shown that, in the absence of nicotine, cigarette smoke is also a modulator of ANS activity, realizing its parasympathetic potential through gaseous and solid-phase components from the first minutes after smoking a cigarette and retaining its effect for several hours. The importance of this conclusion is fundamental in nature, first of all, for those smokers who combine the smoking of regular cigarettes with smokeless nicotine products, as well as for non-smokers using ANDS. The difference between the two types of nicotine users (smokers and non-smokers) is that a smoker (particularly, a frequent smoker) experiences a parasympathetic effect of smoke from each previously smoked cigarette, persisting for a long time (more than 3 h), that weakens the acute sympathetic response of nicotine from next cigarette smoked next, while, in non-smoking ANDS users, in the absence of a modulating and systemic effect of smoke on the parasympathetic division, nicotine in the same doses can affect the ANS in a completely different way. The relevant details of such an influence are considered below.

### 3.2. Comparative Testing Nicotine-Containing Products with Analysis of HRV Changes

Considering the reaction rate and the overall duration of the effect of nicotine-free smoke on ANS activity, it is interesting to compare the effects that occur in a smoker’s body when using different nicotine carriers. One of the smoking volunteers agreed to undergo a trial, the essence of which was to alternate the use of different nicotine delivery systems with long-term HRV tracking by first-lead electrocardiography. Three weeks before the start of the test, the volunteer had the opportunity to test all the products in a trial mode and to work out the correct technique for their use. All trials were conducted on different days in the morning with a 3-h period of abstinence from smoking and eating. The aim of the experiment was to optimize the research protocol and find out the effect of ANDS on HRV in comparison with that of a regular cigarette.

**Figure 7:**
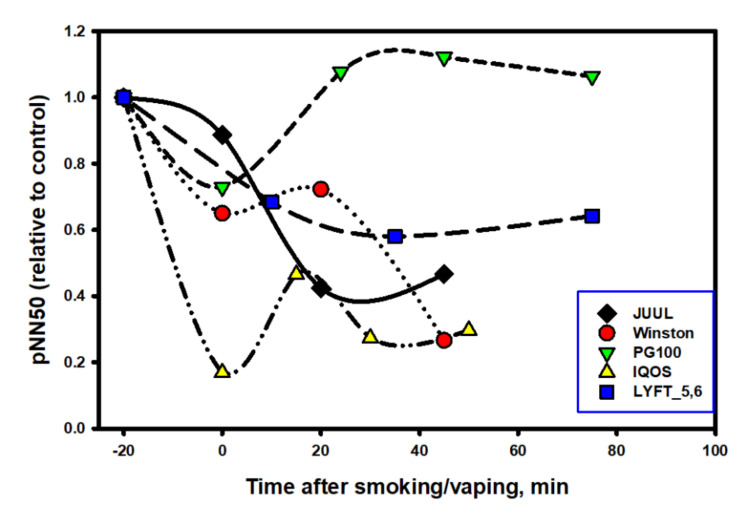
HRV dynamics (percentage of adjacent RR intervals differing by more than 50 ms) after the use of different nicotine-containing products by a smoking volunteer. Electronic cigarette with a nicotine-free filler (100% propylene glycol) was used as a control. The subject took 8 puffs in 3.5 min for all smoking simulators, including cigarettes, and also used a nicotine pack for 40 min.

**Figure 8:**
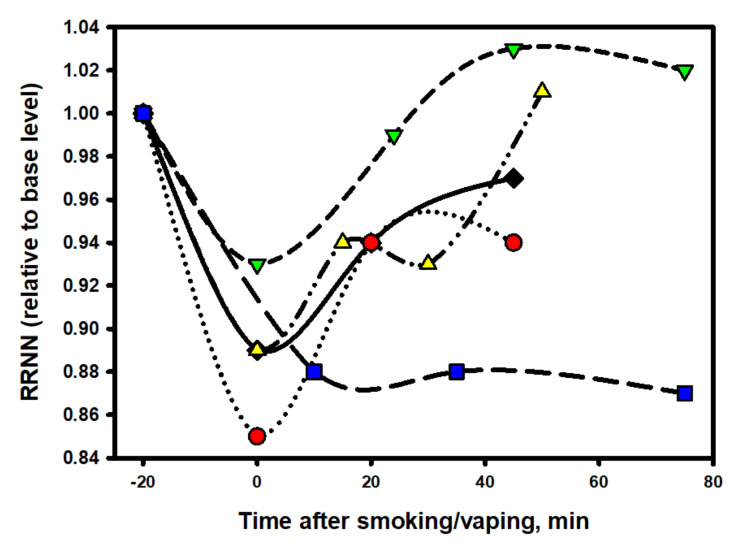
Dynamics of RR intervals after the use of various nicotine-containing products by a smoking volunteer.

From the data exposed in Figure 7 and Figure 8, it follows that each nicotine product had a specific effect on ANS, which was especially noticeable in the first minutes of using the product. Thus, for example, immediately after smoking an ordinary cigarette (Winston brand), the heart rate increased most sharply in comparison with the other options, and the increased heart tone persisted for 45 min. The surge in cardiac activity when smoking a traditional cigarette closely correlates with the release of the neurohormone norepinephrine [48].

Interestingly, the e-cigarette aerosol, consisting of 100% propylene glycol, also slightly increased the heart rate and activity of the sympathetic division of the ANS at the time of the vaping session and immediately after its end, but its effect was short-lived, and after 20 min, the heart rate and balance of the ANS completely recovered to the original level. The most prolonged stimulating effects of nicotine on the cardiovascular system and on ANS persisted when using oral packs, which is understandable, taking into account the long period of the pack fixation in the oral cavity (40 min). However, even after removing the pack, an increased heart rate persisted for at least another 30 min, which was completely uncommon for inhaled forms of nicotine consumption, whether a classic cigarette or an electronic one. This suggests that the dynamics of absorption (and, accordingly, the pharmacokinetics) of nicotine in pack users is different from that which takes place in the case of using inhalation nicotine delivery systems.

The designations of the data points are the same as in Figure 7.

**Figure 9:**
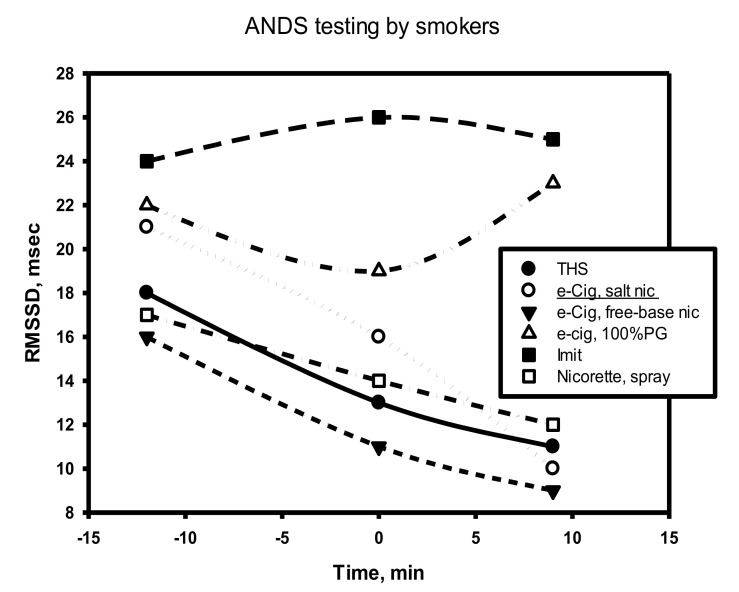
Dynamics of the RMSSD indicator during the period using ANDS by a smoker, including 5 min before the smoking session, the smoking session itself and immediately after its end (usually less than 1 min).

It is noteworthy that, in comparison with nicotine-free smoke, nicotine products that were tested by a volunteer from among the episodic smokers (two to six cigarettes per week) exhibited a completely different and very characteristic effect on the dynamics of RMSSD in the process of the direct use of the product (Figure 9).

As follows from the data in Figure 9, at the time of using any nicotine product (central points on the graph), as well as immediately after the end of its use, the RMSSD indicator always decreased. This indicates a decrease in parasympathetic activity in the subject’s body and the stimulation of sympathetic activity, including central departments. That is, comparing the effects of nicotine-free smoke over a short period of time with the effects of nicotine in different forms of its delivery, one can come to an unambiguous conclusion that smoke and nicotine impose completely different effects (in opposite directions and at different rates) on parasympathetic activity.

**Figure 10:**
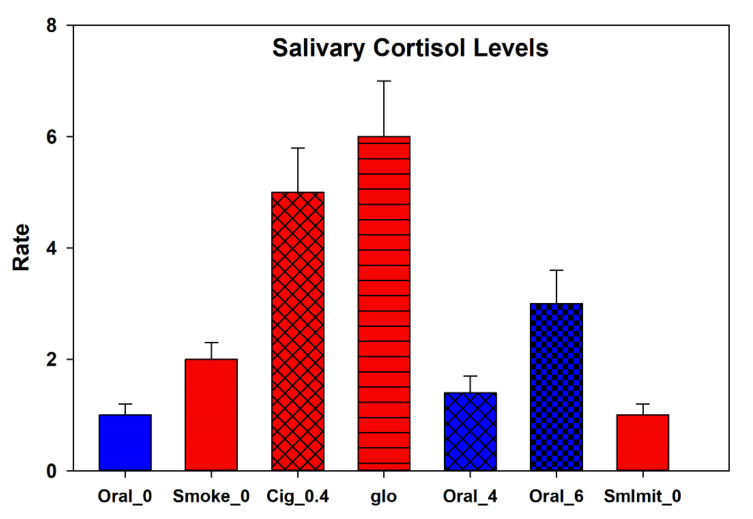
The maximum changes in the level of cortisol (relative to the baseline) in the saliva of a volunteer after testing different types of nicotine and nicotine-free products and the imitation of smoking. Experimental data acquired with oral nicotine products (packs with a nicotine content of 0.4 and 6 mg) are given in blue, while the data obtained for products with an inhalation mod for the nicotine delivery are marked in red (CC refers to a conventional cigarette with nicotine 0.4 mg, glo denotes the glo^TM^ tobacco heating system and a nicotine-free cigarette is designated as Smoke_0, while SmImit is for the imitation of smoking with an unlit cigarette). Data are presented as means of three measurements with the standard deviation. All experiments were carried out at the same time of the day with an interval of 2 to 3 days between tests. The experiment involved a volunteer from the group of rare smokers (2–4 cigarettes per week with a nicotine content of 0.4 mg).

Simultaneously with testing HRV using different nicotine products and nicotine-free smoking simulators, the level of stress hormones was determined in the volunteers. In particular, the results for the analysis of cortisol in saliva are depicted in Figure 10. The test samples caused an increase in the level of this stress hormone an hour and a half after the end of testing the product. The highest relative changes in the cortisol levels among the ANDS tested were caused by the tobacco heating system (more than six-fold increase), while the smallest changes were observed when testing a pack with a nicotine content of 4 mg. In fact, for this pack, there were no significant changes in the hormone levels at all, which is not the case for a nicotine-free cigarette. Even in the absence of nicotine, smoking for a short time, but nonetheless significantly (two times), increased the level of the stress hormone. When comparing the concentration of cortisol in saliva with the data on the analysis of HRV, we came to the conclusion that there is a direct correlation between the LF/HF ratio and the level of the hormone in saliva (r = 0.84, *p* = 0.018), but this rule did not apply to nicotine packs, in which we could not find any close relationship between the cortisol levels and HRV.

In the analysis of the influence of the tested products on ANS, two notable points were evident. Immediately after the use of the tobacco heating system (THS), the HRV decreased sharply (−80%), while immediately after using the EC with a high concentration of nicotine (JUUL), the indicator initially changed little, although the HR dynamics immediately after using both products were almost the same. The smoothest (softest) dynamics of the HRV change were recorded in the experiment with a nicotine pack. This was despite the fact that the HR remained at an increased level for the entire period of the pack fixation in the oral cavity and for at least 20 min after the pack was removed. A close relationship between the HR and HRV was maintained throughout testing the product only in the experiment with the nic-pack. It was characteristic that, for all tested products, the HRV indices were restored much later (with a 20–50-min delay) than the moment of complete recovery of the heart rate.

One of the reasons for the observed differences in ANS reactivity could be the different hydrophilicity of the salt and the conventional synthetic (free-base) nicotine, which determines the rate of its penetration into cells (neurons). Salt (protonated) nicotine, being more hydrophilic and physiological in terms of the pH, on the one hand, is closest in its properties to cigarette nicotine, and on the other hand, it binds worse (slower) to nicotinic receptors and diffuses through membranes than nicotine in its free form [49]. This circumstance largely determines the reaction rate of the nervous system to various forms of nicotine. In ordinary cigarette smoke, the proportion of free (non-protonated) nicotine is 13–15% of the total nicotine content, which, for example, is almost identical to this proportion in the IQOS tobacco heating system [50]. Apparently, for this reason, the profile of the HRV indicator dynamics was similar when using the tobacco heating system and a regular cigarette. The e-cigarette contained 100% saline nicotine, and this fact alone is capable of making significant adjustments to the reactivity of the ANS.

If we take the nicotine-free e-cigarette out of the scope, then among all the nicotine-containing products, the least stress (the least decrease in HRV) was caused by the use of a nicotine pack. Apparently, a smooth increase in the concentration of nicotine in the blood plays a decisive role in the rapid adaptation of the body to vegetative stress. The situation can be compared to two short- and extra-long-distance runners. The first one starts immediately with a jerk without warm-up, while the marathon runner starts calmly and has time to warm up along the course. However, what will happen to the ANS when nic-packs are used systematically? Below, we address this issue.

### 3.3. Testing Nicotine Packs with Systemic Use

For the study, we chose nic-packs with a nicotine content of 4 mg (analog of Nicorette chewing gum with 4 mg of nicotine). A volunteer, a former smoker, agreed to take part in the experiment that involved the use of four packs in 6 h, according to the 45-min scheme, fixing a pack in the oral cavity between the upper gum and lip with a 45-min recovery (without a pack). Before the start of the experiment, during it and at its end, venous blood and saliva were taken to control the level of cotinine and nicotine, as well as other biomarkers. During the experiment, the blood pressure, blood sugar levels and the concentrations of nicotine and cotinine in the saliva were monitored continuously, and an electrocardiogram was taken at 6–8-min intervals for the subsequent HRV analysis. During the experiment, the subject did not have meals, and only drinking pure water no more than three times (150 mL each) was permitted.

**Figure 11:**
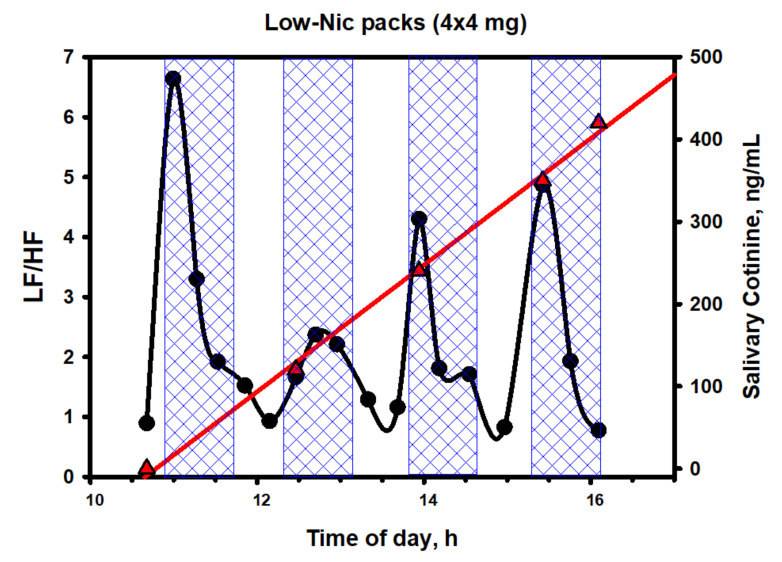
Dynamics of cotinine in saliva (red straight line with triangles) and the LF/HF ratio (black line with circles) during the period of nic-pack testing (4 packs with 4 mg of nicotine). Periods of fixation of packs in the oral cavity are marked with blue-shaded areas.

**Figure 12:**
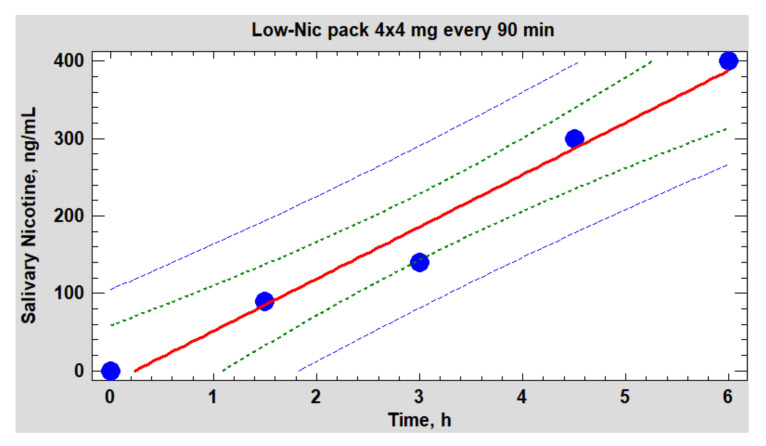
Dynamics of nicotine in saliva (collection of saliva every 45 min immediately before using the next pack). Regression line (red) *p* = 0.0018. 95% confidence level (green dots).

Figure 11 shows the dynamics of cotinine in saliva and that of the LF/HF ratio, while Figure 12 depicts the dynamics of salivary nicotine.

The simplest and most commonly used indicator of epy ANS balance in a spectral analysis is the LF/HF ratio. The data in Figure 11 clearly show that the dynamics of the balance has little in common with the dynamics of either cotinine or nicotine in saliva. However, there is one important nuance. The subject’s sympathetic activity sharply increased immediately after the beginning of the use of the nicotine pack (contact with the mucous membrane of the upper gums), following by a quick decrease to the normal (baseline) values at the local maxima of the nicotine content in saliva and the body as a whole. Preliminary studies have shown that, during the period of the pack fixation on the upper gums, the concentration of nicotine in the plasma reached a maximum only after 30–40 min—that is, in fact, by the time the pack is removed from the oral cavity (Figure 12). However, it was during these periods that the balance of the ANS was completely restored to its original level, which fundamentally distinguishes this product from inhalation tools of nicotine delivery. It seems that the volunteer’s ANS did not react at all to an increase in the concentration of nicotine in the blood but to some other factors, possibly of a psychosomatic nature. The studied phenomenon was not unique to this volunteer, as it was similar, with some variations, in other individuals, mainly former smokers.

The data reported in this part of the work point also to the following important feature of the action of the considered ANDS. The performed spectral analysis of HRV implied that a sharp surge in the activity of the sympathetic division of the ANS (and, accordingly, an increase in the LF/HF ratio), observed within the first minutes of using the nicotine pack, is associated with signal transmission to the brain from nicotinic and taste receptors located in the mouth area. This means that nicotine begins to act already at very low doses before entering the blood physically in an essential amount. A gustatory stimulus placed in the mouth acts on the cilia, triggering a nerve impulse in nearby nerve fibers that are connected to the cranial nerves responsible for taste (facial and glossopharyngeal nerves). Such an impulse moves along these cranial nerves to the brain, which interprets the combination of impulses from different types of taste buds.

**Figure 13:**
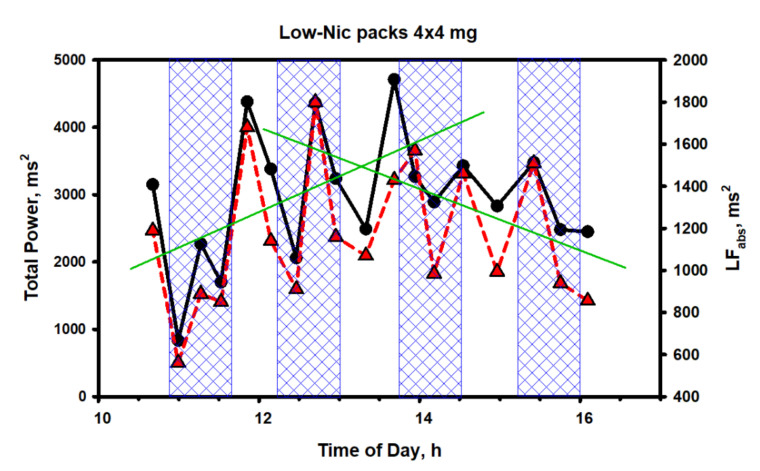
Dynamics of the HRV indicators during testing of the series of 4 nicotine packs (4 mg of nicotine each). The solid black line is the dynamics of the total spectrum power (TP), the red dotted line refers to the dynamics of the low-frequency spectrum component (LFabs) and the green lines are the averaged trend lines of both indicators at the two time intervals (in the first and the second halves of the test cycle).

Continuing with the HRV analysis, a number of interesting points merit attention (Figure 13).

As it is seen from Figure 13, the fluctuations of the two spectrum indicators characterizing the general and sympathetic activity of the ANS also occurred in a very wide range of values, without any reference to the concentration of nicotine in the body. This was particularly noticeable at the initial stage of testing (the first pack and the first shaded area), when nicotine, in fact, had not even entered the bloodstream or was present in ultra-low concentrations, but HRV, closely related to the total spectrum power and ANS activity, decreased to abnormally low values due to activation of the central regulatory mechanisms. Then, an even more interesting feature was observed. The trends of both indicators grew simultaneously during the entire first half of the test (the first and the second packs); after which, they sharply changed direction to the opposite one. Evidently, having experienced initial stress from irritation of the gums at the place of fixing the pack or for some other reason, the body gradually adapted to the new and unusual form of nicotine consumption.

**Figure 14:**
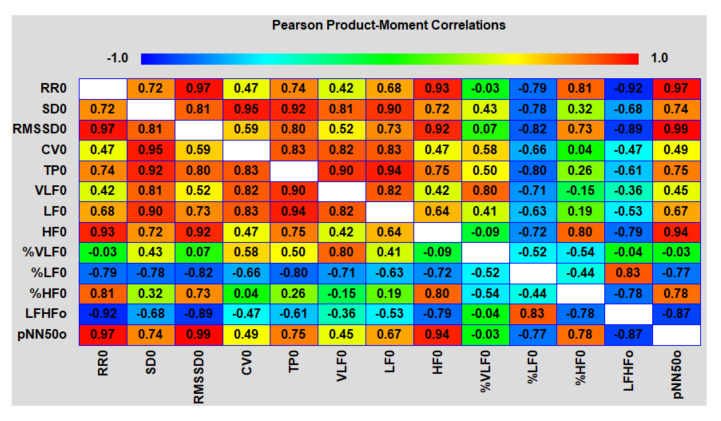
Correlation matrix between the HRV indices at the initial stage (with the descriptor 0 at the end) of the test packs in the interval of 0–3 h (up to the point of intersection of the two green straight trend lines in Figure 14, corresponding to 13 h 30 min).

**Figure 15:**
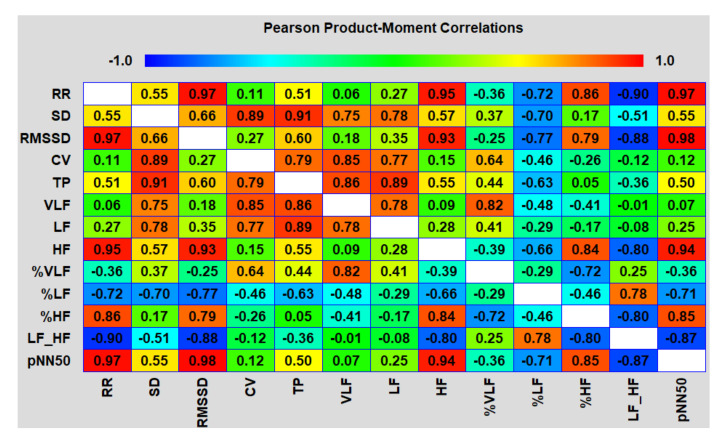
Correlation matrix of HRV indicators at the final stage of the test from the point of intersection of the two green straight trend lines in Figure 14 to the end of using the last pack.

The analysis of the correlation matrices between different HRV indicators in both areas showed an interesting picture (Figure 14 and Figure 15).

**Figure 16:**
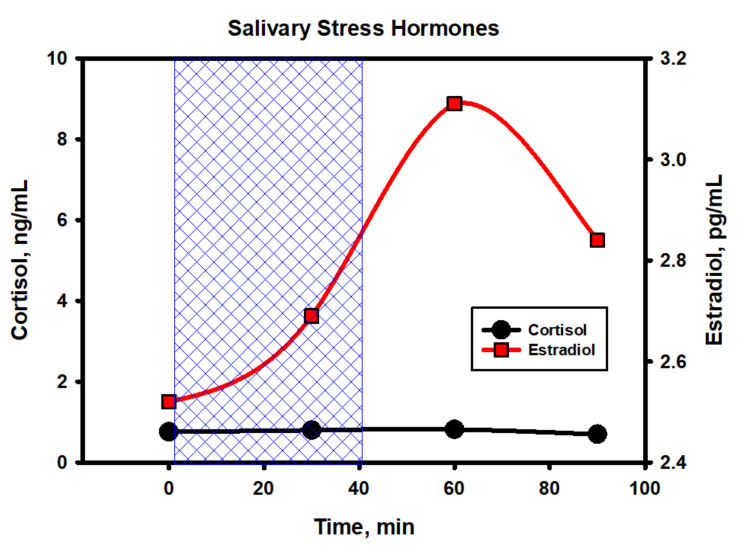
Fluctuations of the stress hormones cortisol and estradiol levels in the saliva of a volunteer during the testing period of a 4-mg nicotine pack. The upper limits of the physiological norms of both hormones, taking into account the time of day, gender and age of the subject, are 2.2 ng/mL and 4.6 pg/mL for cortisol and estradiol, respectively.

The main and, to some extent, unexpected result of the correlation analysis was that both on an upward and downward trend during the testing period of the packs, the duration of epy RR intervals (and reciprocal heart rate) was controlled mainly by the parasympathetic system (correlation coefficients with HFabs, RMSSD and pNN50 are close to 1.0) and, to a much lesser extent, sympathetic, which proves the close relationship of the pharmacokinetics (PK) of nicotine mainly with vagal stimulation. If, when smoking, nicotine reaches its peak concentrations in the blood within 5–7 min from the moment of the first puff of a cigarette, then, when using oral fixation packs with a moderate nicotine content, much more time is required (about 30–40 min). A smooth increase in the level of nicotine in the body avoids a sharp jump in the sympathetic activity (stress of the adrenal glands) and, as a result, prevents the abnormal release of stress hormones in the blood and saliva (Figure 16). From Figure 16, it follows that a pack with 4 mg of nicotine caused minimal fluctuations in estradiol and, in particular, cortisol in saliva within an hour and a half from the beginning of its use. The hormone levels were measured in the afternoon from 14:30 to 16:00, when the natural backgrounds of hormones and, accordingly, the absolute fluctuations are minimal. As shown by the analysis of these hormones, their fluctuations against the background of exposure to the nicotine pack were within the physiological norm, which indicated the minimal physiological stress in the subject during the test period.

Summing up the intermediate result of this experiment, it should be emphasized that the ANS balance, disturbed by the systemic use of oral packs with moderate nicotine contents, tends to recover quickly and completely without any connection with the nicotine content in the body. This indicates the absence of negative systemic changes in the functioning ANS, despite the fact that the total amount of nicotine that entered the subject’s body during 6 h of the experiment could theoretically reach 16 mg (in fact, usually no more than 75% of the nominal nicotine amount in packs), which is equivalent to smoking a package of cigarettes with moderate nicotine content. However, one should make sure that other indicators do not go beyond the physiological norm.

It is known that nicotine and smoking can affect the cardiovascular system by increasing the blood pressure indirectly through the activation of the sympathetic division of ANS. The mechanisms underlying the acute sympathomimetic and pressor effects of nicotine are intricate. Nicotine has a direct pharmacological effect on peripheral post-ganglionic sympathetic nerve endings, which leads to an increase in the release of norepinephrine. The released norepinephrine interacts with β-adrenergic receptors in the cardiac tissue, causing an increase in the heart rate and contractility. Similarly, in vascular tissue, norepinephrine binds to α-adrenergic receptors, causing vasoconstriction. On the contrary, in the brain, acting on sympathetic nerve endings—also indirectly through the release of norepinephrine—nicotine dilates blood vessels by a NO-dependent mechanism [51].

It has been established that the postganglionic excitation of peripheral sympathetic nerves can be suppressed in young people, in whom the pressor effect, mediated by the release of exocytotic norepinephrine, activates regulatory baroreflexes in a negative feedback loop [52]. Baroreflexes have an acute sympathetic inhibitory effect, thereby restoring the blood pressure to its normal level [53]. Studies in smokers have confirmed that activation of the baroreflex by a pressor response masks the increase in postganglionic sympathetic nerve movement after cigarette smoking [54]. However, in aged smokers with weakened baroreflexes, smoking is accompanied by an increase in sympathetic traffic [55]. Thus, while the sympathetic arousal effects of smoking may be reflexively suppressed in healthy young adults with intact baroreflexes, sympathoadrenal arousal may be excessive (and, thereby, potentially lethal) in older smokers with impaired baroreflex function.

**Figure 17:**
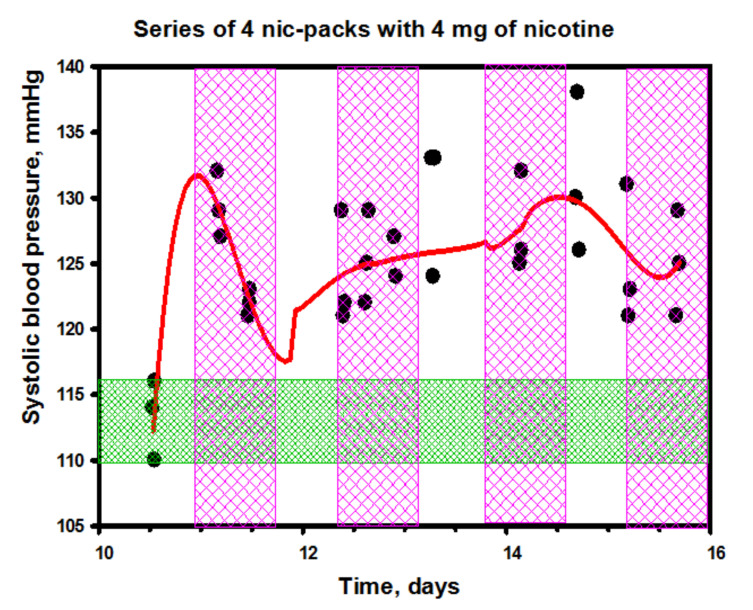
Changes in systolic blood pressure (SBP) during the period of nic-pack testing.

**Figure 18:**
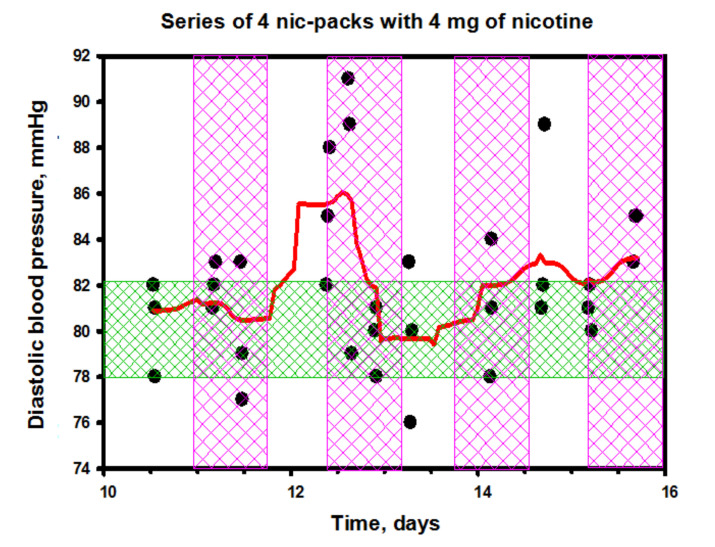
Changes in diastolic blood pressure (DBP) during the period of nic-pack testing.

Relying only on the close relationship between the intensity of sympathetic arousal and baroreflexes, it is difficult to rationalize the observed pattern of changes in the blood pressure (BP) in the subject while using nicotine packs (Figure 17 and Figure 18). Given that the LF/HF balance quickly recovered to the baseline after the initial sharp spikes, one could assume a normal baroreflex function. Moreover, as can be seen from the data of Figure 17, the systolic blood pressure (SBP) after an instantaneous start-up jump also quickly decreased, although a complete return to the initial level did not follow in the future in the complete absence of correlations between the SBP dynamics and the HRV index dynamics. Interestingly, the diastolic blood pressure (DBP) remained practically unchanged (Figure 18).

It is possible that the answer to the question about the reasons for the growth of exclusively systolic blood pressure should be sought in hormones. We studied the dynamics of some hormones in the blood of this volunteer in response to the use of a nic-pack with even a slightly higher nicotine content (5.6 mg) than was used in the previous experiment (Table 1). Characteristically, in addition to a sharp increase in the dopamine levels and a moderate increase in serotonin, no other abnormal or significant changes occurred. The fluctuations in norepinephrine were not as high as expected, and the adrenaline levels were generally stable. It is noteworthy that the level of adrenocorticotropic hormone (ACTH) also did not change during the first hour of using the pack. It appears that the hypothalamus–pituitary–adrenal axis was minimally activated in response to exposure to nicotine in this form of consumption.

A sharp jump in dopamine is of another nature. Dopamine, along with serotonin, could play an important physiological and multifunctional role. The release of dopamine is known to be closely related to the activation of the nicotinic cholinergic system [56]. Dopamine causes an increase in peripheral vascular resistance (less strong than under the influence of norepinephrine) and, exclusively, systolic blood pressure as a result of the stimulation of α-adrenergic receptors without affecting the diastolic pressure. It also moderately increases the force of heart contractions through the mechanism of stimulation of β-adrenergic receptors while having a hypotensive effect [57].

As a neurotransmitter, dopamine is one of the chemical internal reward factors (IRF) and serves as an important part of the brain’s “reward system”, as it induces a feeling of “expectation of pleasure” (or satisfaction), in parallel influencing the processes of motivation and learning [58]. Dopamine is naturally produced in large quantities during positive, in the subjective perception of a person, experiences—for example, from eating tasty foods, pleasant bodily sensations, etc. [59]. Its release signals a pleasant experience to the brain and is critical to the development of nicotine addiction or the onset of quitting smoking. However, dopamine (which is formed in the adrenal glands and kidneys with circulation in the blood) and the dopamine neurotransmitter (which is formed in the brain) are, in fact, little or not-related concepts. Due to the properties of the blood–brain barrier, the circulation of large amounts of dopamine in the blood has little effect on its level in the brain and vice versa. According to some data, a minor part of blood dopamine comes from the nervous system, another 2% is the contribution of the adrenal glands [60]. Thus, the question arises, where is so much dopamine in the blood generated by the action of nicotine? How is this process related to the vegetative activity?

Human plasma contains three endogenous catecholamines, namely, dopamine (DA), norepinephrine (NE) and epinephrine (EPI). NE in the bloodstream originates primarily from networks of sympathetic nerves that span blood vessels throughout the body and penetrate organs such as the heart and kidneys. Thus, plasma NE concentrations are widely used to measure the activity of the sympathetic nervous system. Plasma EPI concentrations usually reflect the neural outflow to the adrenal medulla and subsequent secretion of adrenomedullary chromaffin cells into the bloodstream.

While the sources of plasma NE and EPI are well-known, the sources of the third endogenous catecholamine, DA, remain poorly understood. Normal DA concentrations in the plasma are usually very low, about 0.1 nM (or about 15 pg/mL) [60]. A significant part of the dopamine entering the circulation is formed in the gastrointestinal tract when food is broken down. Plasma DA increases after a standard meal and decreases after prolonged fasting [61]. Taking in a standard meal increases the plasma concentration of DA sulfate by more than 50 times. The liver removes and metabolizes at a high rate all the catecholamine delivered to it through the portal vein. Thus, although the gastrointestinal tract is the main site of DA production in the body [62], a very small part of free DA in the systemic circulation comes from the intestine.

Another potential source of DA in plasma is non-neuronal uptake and decarboxylation of circulating dihydroxyphenylalanine (DOPA), catalyzed by L-aromatic amino acid decarboxylase. It is known that this process takes place in the kidneys [63]; however, this contribution is probably not high either.

A possible third source of free (unconjugated) DA in plasma is sympathetic noradrenergic nerves. According to this concept, DA is co-released with NE during exocytosis [64]. If, in our case, DA in the plasma were derived from sympathetic nerves, as suggested by Goldstein et al. [64], a positive correlation between the changes in plasma DA and NE could be predicted. However, in our study, when using nic-packs, such a correlation was absent. From this, it can be concluded that the activation of the sympathetic nervous system did not play a major role in the increase in the level of dopamine in the blood during the period of the nic-pack trial.

Interestingly, as early as 30 years ago, it was shown that the releases of DA and NE do not correlate with each other during sympathetic stimulation [65]. Moreover, it was found that endogenous DA limits the release of catecholamines during sympathetic-adrenal stimulation by activating the DA2 receptors [66].

We believe that, when nicotine enters the body through the oral mucosa, as is the case with the use of a nicotine pack, a part of nicotine enters the gastrointestinal tract, which becomes the main source of an increase in the plasma DA levels. Indirectly, this is indicated by the change in the microbiocenosis in the small intestine of the volunteer during the testing of the nic-packs (Table 2).

The gut microbiome can produce hormones and neurotransmitters identical to those produced by humans. Several studies have shown convincingly that the gut microbiota plays a critical role in the generation of free catecholamines and dopamine [67]. By producing neurohormones, the gut bacteria directly stimulate afferent neurons in the intestinal nervous system by sending signals to the brain via the vagus nerve. Bacteria can synthesize and respond to hormones and neurotransmitters. *Lactobacillus* species produce acetylcholine and gamma-amino butyrate (GABA); *Bifidobacterium* species produce GABA; *Streptococcus* and *Enterococcus* produce serotonin; *Escherichia* produce norepinephrine, serotonin and dopamine and *Bacillus* species produce norepinephrine and dopamine [68]. Xue et al. showed that clearance of the gut microbiota by antibiotic treatment resulted in a reduced synthesis of dopamine in the intestines, and the liver was damaged excessively. That could be corrected by the recovery of gut microbiota or replenishment of the D1-like receptor agonist [69].

We studied the composition of the resident and transit bacterial microflora of the small intestine of a volunteer before and after the application of a series of four nic-packs and came to the conclusion that the composition of the microflora changed little over the 8.5 h of the experiment, except for a small growth of *Clostridium ramosum* (+60%) and *Ruminococcus* spp. (+30%) within the resident group (Table 2). The composition of the fungi also changed little, with the exception of a small growth of micromycetes. It is noteworthy that the synthesis of dopamine by the intestinal microflora is closely related to the ability of certain species and genera of bacteria to produce β-glucuronidase (GUS). Yoneda et al. reported that conjugated catecholamines in peripheral blood can be easily converted to free forms by in vitro incubation with bacterial GUS [70].

Despite the fact that, in the brain, dopamine can be associated with positive psycho-emotional arousal, an increase in the level of dopamine in the blood plasma acts as an antistress factor in shock, trauma, burns, blood loss, with various pain syndromes, anxiety and fear; that is, at moments when the body needs to adapt to stressful situations. Additionally, the level of dopamine in the blood increases with a deterioration in the blood supply to the kidneys or with an increased content of sodium ions, as well as angiotensin or aldosterone in the blood plasma. Apparently, this is due to an increase in the synthesis of dopamine from DOPA in kidney tissue during ischemia or when exposed to angiotensin and aldosterone. However, this pathway is not able to greatly affect the level of DA in plasma.

Considering that, in our experiment with nicotine packs, the levels of stress hormones in the subject’s saliva and blood did not change much, we can conclude that the observed physiological effects (a slight increase in systolic pressure and sharp jumps in the autonomic balance) were mainly associated with the dynamics of dopamine in the blood, which, in a significant amount, appeared in the blood after exposure to nicotine in the gastrointestinal tract.

As an antistress factor, dopamine is nevertheless formed with the direct involvement of the sympathetic nervous system. It can also be said that the psycho-emotional stress response of the subject to nicotine packs was somewhat ahead of the physiological effects of nicotine in terms of time. Obviously, a smoker with experience over many years of smoking cigarettes develops a mental reflex to the rapid supply of nicotine to the brain associated with smoking a cigarette, and this reflex is partially reproduced even with a slow increase in the concentration of nicotine with completely different forms of its delivery. In this case, it is appropriate to talk about the effect of small doses of nicotine, which triggered the vegetative activity in the first minutes of contact of the pack with the mucous membrane. We already mentioned this phenomenon while discussing the data exposed in Figure 11.

The role of dopamine and dopamine receptors in modulation of the ANS has been studied in detail in rats [71]. Stimulation of the serotonergic system caused a significant increase in the heart rate and a decrease in the amplitude of all waves of the HRV frequency spectrum. Stimulation of the dopaminergic system led to a moderate increase in the heart rate and amplitudes of low-frequency (LF) and very low-frequency (VLF) HRV waves. The authors suggested that the serotonin and dopaminergic systems affect the heart rate through the receptors of cardiomyocytes and through the modulation of the activity of the adrenergic and cholinergic systems. The effects of the serotonin and dopaminergic systems can be viewed as synergistic in the central nervous system and antagonistic in the periphery. By blocking nicotinic receptors, the authors found that the dopamine stimulation modulated HRV exclusively through peripheral mechanisms, while serotonin acted at the humoral and neuronal levels, involving systemic and local mechanisms.

## 4. Discussion

For the discussion of the results disclosed in the present work, it is noteworthy that previous studies on the considered subject were mostly of fragmentary character and did not take into account the intricacies derived from the influence of a number of physiologically active components entering the living organism along with nicotine when smoking or using ANDS. Most prominently, this refers to the influence of carbon monoxide, the examining of which constitutes the major challenge. In the reported work, we addressed this important issue. In this context, the development and verification of a method for testing nicotine-free smoke are of great practical significance for predicting short- and long-term health consequences for smokers after quitting smoking and using nicotine in general, on the one hand, and with partial or complete cessation of using conventional cigarettes in favor of ANDS, on the other. First, many components of smoke have a longer residence time in the body than nicotine. For example, if the half-life of nicotine in the body of a smoker or user of ADNS amounts to minutes or tens of minutes, then carboxyhemoglobin (COHb), as a product of the reaction of hemoglobin with the CO of smoke, the decomposition of which leads to an increase in CO in the exhalation, lingers in the body for several hours. The terminal half-life of COHb (3.47–5.97 h) depends on the duration of CO exposure, CO concentration, peak COHb concentrations and individual parameters of the smoker [72]. According to other data [73], the half-life of carboxyhemoglobin when toxic levels are reached is somewhat shorter and amounts to 1.23–2.28 h. The level of carboxyhemoglobin plays an important role both in the diagnosis of a number of diseases and in assessing the impact of combustion products, including smoke chemicals.

The physiological role of CO and carboxyhemoglobin is still not well-understood. It is known that, in acute carbon monoxide poisoning, the heart and the brain are the first to be affected, as the organs most sensitive to hypoxia. In relatively healthy people living in ecologically favorable regions, the COHb level usually does not exceed 1% and is a consequence of the endogenous origin of CO. According to various estimates, the COHb level from 3% to 5% is the threshold and is usually not accompanied by toxic effects, while the range of 10–20% refers to moderate carbon monoxide poisoning and may be accompanied by headache, lethargy or fatigue. Acute CO toxicity is manifested by a decrease in the loss of consciousness, a sharp decrease in the blood pressure and bradycardia up to cardiac arrest. Interestingly, smokers are long-term exposed to elevated levels of carbon monoxide in cigarette smoke, and healthy heavy smokers can tolerate carboxyhemoglobin levels up to 15% or more without any problems.

A smoker with many years of experience gradually develops a tolerance, adaptation and, in some ways, synchronization of the body’s response to the actions of nicotine and CO in a certain proportion. In the cases of a sharp cancellation of the CO intake (as can occur with a complete or partial cessation of smoking in favor of ANDS) or a sharp increase in the intake of nicotine (for example, with the simultaneous use of conventional cigarettes and ANDS), negative reactions in the smoker’s body may occur. It has been found that partial cessation of smoking and switching to ANDS in some smokers causes hypertension and arrhythmia and increases the frequency of asthmatic attacks and the risk of developing diabetes mellitus, as well as other negative health effects [74]. Although the level of carboxyhemoglobin was not controlled in this experiment, it is likely that some of these exacerbations of diseases are associated with a sharp change in the usual ratio of levels of carboxyhemoglobin and nicotine towards the dominance of the latter. If CO has parasympathetic stimulation and nicotine is sympathetic (in the first minutes of intake), then a change in the ratio of these agents in the body can change the vegetative balance and affect the health. Paradoxically, one of the reasons why active smokers have a lower risk of hypertension than former smokers and non-smokers is associated precisely with the increased level of carboxyhemoglobin in the blood of regular smokers [75].

Taking into account the fact that smokers often develop COPD (often with an asthmatic component), it is interesting to look at the relationship of pathogenesis with functioning ANS and the role of exogenous carbon monoxide in this process (in the composition of cigarette smoke). A number of studies have shown that the irritation of nerve receptors in the lungs causes stimulation of the parasympathetic division of the ANS. Thus, Nadel [76] and Gold [77] accumulated evidence that the stimulation of rapidly adapting epithelial nerve receptors of the airways by mechanical, chemical and pharmacologic stimuli increases the output of acetylcholine by the vagus nerves by reflex, causing a reflex bronchoconstriction [76,77]. Probably, this is precisely the explanation of the reason why asthmatics have higher parasympathetic activity than healthy people [78].

Thus, smoke inhalation should be accompanied by a change in HRV and an increase in parasympathetic activity indicators. To verify that, we conducted an experiment to investigate the effect of smoking a nicotine-free cigarette on HRV and ANS balance, which confirmed the initial assumptions.

The idea of creating a variety of ANDS logically resulted from a deep study of the composition and properties of cigarette smoke and an understanding of the role of the individual components of smoke in initiating various pathologies characteristics of long-term smokers. The known fact that nicotine in its “pure” form does not have such physiological side effects as nicotine in cigarette smoke inspires optimism concerning the significant reduction of smoking. Nevertheless, it is still not fully clear what form of smoking cessation is the safest for smokers. The active promotion of ANDS among smokers as low-risk products has stimulated a surge of interest in these products, the result of which is that many smokers have become users of both traditional cigarettes and ANDS. Numerous literature data indicate that smokers from the dual-user category are often at greater risk of disease than only smokers or only ANDS users. However, the exact reasons for this phenomenon are still poorly understood. The results of our study clearly show that macrocomponents of smoke can have a multidirectional effect on the ANS. In the absence of nicotine, smoke stimulates the parasympathetic activity, which, however, cannot be considered as a favorable (antistress) factor, since an increase in the parasympathetic activity (as well as sympathetic activity) can be a consequence of chemical or physical stimulation of the nerve receptors of the lungs by various smoke toxicants (particles, tar constituents and gaseous combustion products) even in relatively low concentrations. For this reason, a change in the balance of smoke components by the additional intake of smoking nicotine into the body can significantly aggravate the situation and increase the health risks. In this context, the use of nicotine packs is a good alternative to both regular cigarettes and many ANDS (including snus), primarily because it allows one to slowly and, for a long time (up to 45 min or more), satisfy a smoker’s need for nicotine with a minimal content of all kinds of impurities.

The pharmacodynamics of nicotine, like many other psychoactive substances, is largely determined by its pharmacokinetics. Nicotine in oral form is slowly released, which allows avoiding an instant jump in its level in the blood and brain, as is usually observed after smoking cigarettes, using electronic cigarettes and tobacco heating systems. A recent study with healthy smokers showed that the blood pressure, but not heart rate, can increase even after using nicotine-free e-cigarettes [79], which is very difficult to explain from a purely physiological point of view. It is possible that psychosomatics plays a certain role in the effects associated with the use of nicotine products and nicotine-free smoking simulators, and the HRV analysis is a good tool for studying these phenomena.

In view of the linkage between the activity of the ANS and the hormonal system [6], in the present work, the analysis of the key hormones as a function of consuming diverse nicotine products was carried out.

Influencing the activity of the sympathetic part of the ANS, nicotine stimulates the release of catecholamines, such as norepinephrine, but, at the same time, nicotine is primarily cholinomimetic in nature, and nicotinic nAChR receptors are widely and densely distributed throughout the body. Another aspect of the effect of nicotine on the body is associated with the fact that it has a bimodal dose-dependent effect on almost all organs at the ganglionic level. At low doses, it stimulates the autonomic ganglia and facilitates impulse transmission, whereas, at higher doses, the initial stimulation is followed by a ganglion block.

The highest relative changes in the levels of stress hormone cortisol among the ANDS tested have been observed for the case of the tobacco heating system, while only nominal changes in the cortisol level were caused by nic-packs with a nicotine content of 4 mg.

Examining, for the first time, the influence of nicotine-free smoke on the cortisol level has shown that smoke, for a short time but, nonetheless, significantly, increases the level of such a stress hormone.

Comparing the concentration of cortisol in saliva with the HRV data revealed a direct correlation between the LF/HF ratio and the level of the hormone in saliva, but this does not apply to nicotine packs in which we could not find any close relationship between the cortisol levels and the HRV characteristics.

Currently, there are ongoing debates about the role of nicotine in the pathophysiology of atherosclerosis and whether it can accelerate vascular disease. There are several mechanisms for the negative effect of nicotine on the cardiovascular system. It can cause strong sympathomimetic effects, decrease coronary blood flow, disrupt endothelial function, increase inflammation and arteriogenesis and induce insulin resistance [80]. Epidemiological data that are consistent with these results are scarce, as most people tend to use tobacco products rather than nicotine-only products. Thus, there is no reason to look for causal relationships and equate the effects caused by smoking or using other tobacco products and those that occur solely due to the “fault” of nicotine.

The nicotine packs studied in our work have been shown to be the type of product that can be safely attributed to one of the safest and most convenient forms of nicotine consumption, with the only amendment that the nicotine content in them does not exceed the reasonable limits. Another important conclusion is that nicotine, even in the most convenient and purified form from toxic impurities, still remains a pharmacologically active compound that activates a huge number of receptors throughout the body, modifying a variety of functions. By studying the work of the ANS, as was done in our study, and combining it with the usual clinical practice of examinations, it is possible to assess the consequences of using a new type of product at a personal level faster and more efficiently, taking into account the history of smoking, propensity to develop diseases, etc.

The relatively long history of the research on using snus provides valuable experiences for predicting the risks associated with other oral nicotine products, such as tobacco-free nicotine packs, which have only recently entered the global and domestic markets. Although snus users have a lower risk of lung cancer than smokers, oral tobacco use is associated with increased mortality after myocardial infarction and stroke, as well as an increased risk of type II diabetes [81]. At the same time, it has been shown that transdermal nicotine replacement therapy is apparently safe even for patients with cardiovascular disease [82]. The latter fact inspires some optimism with regards to tobacco-free nicotine packs of oral fixation. The extended in time extraction of nicotine when using nic-packs positively differs from the absorption of nicotine in cases of inhalation forms of its delivery, such as conventional cigarettes, e-cigarettes or tobacco heating systems. On the other hand, unlike snus, nic-packs do not contain tobacco, which should further reduce the biological risks associated with their use.

Finally, monitoring the ANS activity through the HRV measurements constitutes a universal approach to the assessment of risks associated with consuming nicotine products. Another general approach to assess the mentioned risks could be based on the potential of nicotine products to promote the development of oxidative stress, a multifaceted generator of pathologies [83,84,85,86,87,88]. However, the HRV analysis is certainly more facile and preferable on economic grounds.

## 5. Practical Application

The present study forms the fundamentals for the practical application in the assessment of the biological risks of using diverse nicotine products and delivery systems. The HRV analysis constitutes a facile diagnostic tool that combines efficiency, mobility, simplicity, economy, sensitivity and selectivity. As for the limitation of the disclosed methodology, clearly, the HRV analysis does not replace or cancel biochemical monitoring but only complements it, allowing one to concentrate resources on the most problematic areas.

The analysis of the biological impacts of the diverse nicotine products exemplary studied in the present work merits further extension to a wider range of nicotine products—most prominently, to emerging nicotine delivery systems, which rapidly appear on the consumer market. Another potential direction of further research refers to studying the influence of smoking history and behavior on the response of the ANS and hormonal system to diverse ANDS for a more efficient prediction of the risks associated with using different nicotine delivery tools and for developing a rational strategy for smoking cessation.

## 6. Conclusions

The important practical conclusion that can be drawn from the results of our study is that the analysis of the ANS activity within the framework of a smoking cessation program makes it possible to quickly and objectively assess the stress state of the smoker and choose the optimal alternative not only to ordinary but, also, to some electronic cigarettes, which will ultimately increase the chances of forever quitting smoking and reducing the risks of diseases caused by exposure to cigarette smoke.

Based on the results disclosed herein, we conclude that the HRV method is a facile and promising technique for the evaluation of the risks associated with smoking, the dual use of various ANDS and for studying the biomedical aspects of smoking cessation, as it is capable of assessing the impact of the pertinent physiological, toxicological, psycho-emotional and nervous factors. In general, smokeless nicotine products exhibit a reduced impact on humans compared to traditional cigarettes, and nic-packs are shown to be leaders in biological safety among the studied ANDS. A sharp surge in the activity of the sympathetic division of the ANS within the first minutes of the use of nicotine packs implies that nicotine begins to act already at very low doses (before entering the blood physically in any significant amount) through fast signal transmission to the brain from the nicotinic and taste buds located in the mouth area.

## 7. Outlook

The experimental results reported herein could motivate the further in-depth investigation of the toxicological, physiological, biophysical and biomedical aspects of using traditional, novel and emerging nicotine products aimed at predicting the health consequences of their use.

## Figures and Tables

**Table 1:** Dynamics of hormones in the blood during the test period of a 5.6-mg nicotine pack ^a^.

Hormone	0 h (Control)	1 h	2 h	Norm Limits	
				Min	Max
Adrenaline, pg/mL	42	47	46	0	110
Noradrenaline, pg/mL	308	344	395	70	750
Dopamine ^b^, pg/mL	5	334 *	530 *	0	87
Serotonin, ng/mL	136	184	194	50	220
ACTH ^c^, pg/mL	8.5	8.95	8	5	46
STH ^d^, ng/mL	1.44	0.07	0.64	0	8.00

^a^ The experimental errors are within 7%. ^b^ The sum of free and conjugated dopamine. ^c^ ACTH = adrenocorticotropic hormone (both produced in the anterior pituitary gland). ^d^ STH = somatotropic hormone (growth hormone). * very sharp changes.

**Table 2:** Generalized composition of the microflora of the small intestine (10^5^ cells/g) *.

Microorganisms	Before the Experiment	Norm	After the Experiment	% of Change
Residential (a sum)	10,780	19,627	12,837	+19.6
Transitory (a sum)	0	33	0	0
Including				
Anaerobic bacteria	10,476	17,687	12,085	+15.3
Firmicutes	7504	12,085	9305	+61.0
Actinobacteria	3251	7477	3441	+5.8
Bacteroidia, Flavobacteria	0	35	0	0
Proteobacteria	26	63	86	+231
Fungi	1328	2332	2974	+124
Viruses	517	1444	2348	+354
Endotoxin, nmol/mL	0.08	0.5	0.26	+225

* The experimental errors are within 12%.

## Data Availability

Not applicable.

## Supplementary Materials

The following supporting information can be downloaded at https://www.mdpi.com/article/10.3390/biomedicines10010121/s1, Table S1: ECG measurement protocol and the experimental design during the testing period for nicotine-free cigarettes. Table S2: Multiple range tests (for the RMSSD indicator) with and without taking into account the smoking session. Table S3: Estimated difference between each pair of means (Bonferroni procedure). Table S4: Multiple Range Tests. Method: 95% Games-Howell. Table S5: Two-way analysis of variance MANOVA of test results of a series of two nicotine-free cigarettes (“Smoking”) in non-smokers with different initial vegetative status (ANS *). Figure S1: Dynamics of the average group adjusted RMSSD values in the process of testing nicotine-free cigarettes (1C stays for the first cigarette, while 2C refers to the second cigarette) relative to sham smoking (K), depending on the initial ANS type: S refers to sympathotonia (LF/HF > 2.5), N denotes normotonia (LF/HF = 0.7–2.0), while V designates vagotonia (LF/HF < 0.7), a refers to statistically significant difference with control and b pertains to difference with the first cigarette. Figure S2: Dynamics of the group mean adjusted RMSSD values in the process of testing nicotine-free cigarettes (the same designations) relative to sham smoking (K) for all subjects.
