# Supplementary material for: Influence of Nicotine from Diverse Delivery Tools on the Autonomic Nervous and Hormonal Systems
Source: Biomedicines. 2022 Jan 6;10(1):121. doi: 10.3390/biomedicines10010121 (PMC8773565; doi:10.3390/biomedicines10010121)
Supplement: Supplementary file 1 [file biomedicines-10-00121-s001.zip › biomedicines-1472489-supplementary.pdf]

biomedicines

Supplementary Material

Influence of Nicotine from Diverse Delivery Tools on the Autonomic Nervous and Hormonal Systems

Valerii A. Menshov [1,*], Aleksei V. Trofimov [1,2,*], Alla V. Zagurskaya [3], Nadezda G. Berdnikova [1,4], Olga I. Yablonskaya [1] and Anna G. Platonova [5]

1 Emanuel Institute of Biochemical Physics, Russian Academy of Sciences, Moscow, Russia; berdnad@mail.ru (N.G.B.); olga.yablonsky@gmail.com (O.I.Y.)
2 Moscow Institute of Physics and Technology (National Research University), 141701 Dolgoprudny, Russia
3 Medical Center AVC & DNKom Laboratory, 119334 Moscow, Russia; alla.viktorova.2014v@mail.ru
4 I.M. Sechenov First Moscow State Medical University, 119991 Moscow, Russia
5 MedBazis LLC, 190013 Saint Petersburg, Russia; platonova@medbazis.com
* Correspondence: vinoman66@mail.ru (V.A.M.); avt_2003@mail.ru (A.V.T.); Tel.: +7-495-9397358 (A.V.T.); Fax: +7-499-1374101 (V.A.M. & A.V.T.)

Assessment of the air pollution impact through the HRV measurements

In one of these studies, a close relationship between the level of air pollution and the ANS activity was studied in detail and shown in volunteers taking and not taking calcium channel blockers [12]. Typically, possible mechanisms for these associations include an effect on the ANS through direct airway reflexes or an inflammatory response, chemical effects on ion channel function in myocardial cells, ischemic response in the myocardium, and inflammatory responses that cause endothelial dysfunction, atherosclerosis, and thrombosis [13]. Research results by Park et al. [12] showed that a decrease in HRV (increase in the LF/HF ratio) is closely correlated only with air PM2.5 particles and ozone concentrations in case of a combination of factors. Neither CO, nor nitrogen and sulfur oxides exhibited any effect on HRV. The authors conclude that air pollution has the ability to affect both sympathetic and parasympathetic pathways. The sympathetic response seems to be mediated by pathways related to calcium flux into cells, whereas the parasympathetic response seems likely to be due to other mechanisms. The authors do not report what are the exact mechanisms, which lead to the stimulation of the parasympathetic division of the ANS.

It must be recognized that our current knowledge of the pathophysiological mechanisms that link the nature of air pollution and changes in the autonomic nervous system is very limited. One possible mechanism is that inhalation of dust particles induces oxidative stress directly or through the acute pneumonia. Oxidative stress in the respiratory organs causes an increase in the level of proinflammatory mediators that stimulate extracellular calcium influx, possibly due to the activation of calcium channels in the plasma membrane [14] and inactivation of nitric oxide [15]. It is believed that these effects cause an increase in the activity of the sympathetic nervous system and a decrease in the tone of the vagus nerve [16], which, in turn, may be associated with the appearance of ventricular arrhythmias and myocardial dysfunction. In general, the authors agreed that air pollution is more likely to provoke a decrease in vagal tone than an increase in sympathetic activity. However, it is not clear against this background why the parasympathetic activity increases in asthmatics, despite the fact that all signs of inflammation and oxidative stress in the bronchial tree are obvious [17].

According to other authors, combined exposure to moderate levels of ultra-fine particles (UFP) and ozone in the air increases only the bioavailability of peripheral norepinephrine due to a decrease in its clearance, but not due to changes in central vegetative activity [18]. However, a study from the Utah Valley found positive associations between PM10 and RMSSD [19]. Additionally, dogs exposed to concentrated ambient air particles

showed significantly higher HF compared to filtered air exposure [20]. Godleski et al. [20] argued that too much elevation in parasympathetic stimulation may deteriorate cardiac status and result in a fatal bradyarrhythmia.

Protocol for testing nicotine-free cigarettes on non-smoking volunteers

The protocol for testing nicotine-free cigarettes on non-smoking volunteers is given with explanations in Table S1. Volunteers who had never consumed nicotine in any form before took part in the trial of nicotine-free cigarettes. They signed an informed consent to participate in the experiment and transfer the test results to a group of researchers for subsequent processing, storage and publications. In this regard only volunteers whose CO level in exhalation did not exceed 3 ppm were selected for participation in the experiment. Previously, two weeks before the start of the experiment, each volunteer was offered to undergo smoking training in an introductory mode to work out the procedure for testing nicotine-free cigarettes with simultaneous ECG measurement. The experimental procedure and the used hardware-software complex allow testing of smoking devices by volunteers in a self-diagnosis mode without the participation of auxiliary personnel.

Table S1. ECG measurement protocol and the experimental design during the testing period for nicotine-free cigarettes.

<table>
<tr><th>Experiment stage</th><th>Time after start</th><th>Stage duration</th><th>Description</th></tr>
<tr><td>Start. Control HRV measurements</td><td>0:00 (h:min)</td><td>60 min
4 ECG control points</td><td rowspan="4">ECG was taken in a sitting position at intervals of 6–9 min every 20 min. In the intervals between measurements, 5 min of active walking. Then the procedure was repeated. During smoking and simulating smoking, the subject was allowed to move one hand at the level of the head with a fixed electrode to puff a lit or unlit cigarette.</td></tr>
<tr><td>Sham (simulated) smoking</td><td>1:20</td><td>10 min of sham smoking and 60 min of dynamics assessment</td></tr>
<tr><td>Smoking of the first cigarette</td><td>2:00</td><td>10 min of smoking and 80 min of dynamics assessment</td></tr>
<tr><td>Smoking of the second cigarette</td><td>3:40</td><td>10 min of smoking and 80 min of dynamics assessment</td></tr>
</table>

Sampling the biomaterial (saliva and blood) during the test, monitoring the CO content in exhalation and measurement of blood pressure were carried out strictly according to the schedule and usually immediately after measuring the ECG. For cortisol hormone assay saliva was collected using a cotton pledget of Salivette ™ system. Stimulation of salivation was induced by chewing a cotton swab in accordance with the instructions.

The blood pressure was measured with a Hartmann Tensoval Duo Control equipped with a dual sensor technology for pulse tracking. During the entire test cycle, the volunteers were only allowed to drink clean still water in a total amount of no more than 0.5 L. The air temperature in the test room was 23 0C, air exchange in the room was ensured by continuous ventilation. Air purity control in the room, where smoking was taking place with simultaneous ECG measurement was carried out along with continuous monitoring the PM2.5 content, volatile organic pollutants and formaldehyde in the air.

ECG recording while smoking. When assessing the effect of cigarette smoking on ANS activity by the ECG method, several important points and limitations should be taken into account, which were established empirically and are important for avoiding erroneous results:

• It is usually difficult even for an experienced smoker to control the rate and depth of breathing while smoking a cigarette (usually during 5-7 min, including the preparation procedure). It is more difficult to do this while measuring the ECG, when the smoker remains completely immobile or with minimal activity. Therefore, pre-training is required for non-smoking volunteers;

• In volunteers who have never smoked, smoking even a nicotine-free cigarette can cause psycho-emotional stress that can alter the HRV and the ANS balance. After a few training sessions, the stress of smoking a cigarette usually disappears;

• It is difficult to control the depth and duration of each puff while smoking a cigarette, but it is even more difficult for non-smoking volunteers to reproduce the puff profile when simulating smoking in the absence of the real smoke. This problem was again solved by training with the involvement of smokers as consultants;

• Deep breathing has been proven to trigger acetylcholine production in the vagus nerve. It was lowering the heart rate, lowering the blood pressure and relaxing muscles. Exposure to nicotine (at least in the first minutes after ingestion) usually leads to the opposite effect. In this regard, empirically we selected the optimal respiratory rate in experiments with nicotine and nicotine-free smoking simulators at the level of 13–15 breaths per minute for all the participants in the experiment. The subjects independently controlled their breathing cycles using a timer;

• Despite all the technical difficulties associated with obtaining the ECG data while smoking a cigarette or simulating smoking, when testing the properties of smoke, ANS control should be started from the first puff or even a little earlier, when the smoker just takes out a cigarette from the pack and brings it to his mouth for lighting. Already at this moment there is a shift in the psycho-emotional state, which is reflected in the HRV indicators. Nevertheless, taking an ECG in the first minutes before starting smoking and during smoking gives very important information about the state of the ANS and the acute reaction of the body to the effects of smoke and smoking in general.

Primary analysis and processing of experimental data.

The data analysis procedure includes mandatory tests for the normality of samples and the identification of anomalous values (outliers), which are routinely included in the statistical packages used. In addition, when comparing a set of samples (data groups), united by a single concept of the experiment, methods of a posteriori analysis with different strategies with respect to the risk of occurrence of the so-called "type I error" when testing the null hypothesis were used to identify pairwise differences [27]. The choice of both conservative and liberal assessment procedures for analysis was aimed at maximally objectively studying the effect of various smoking simulators on HRV. A smoking simulator in a broad sense is any product similar in design, tactile and organoleptic sensations or functionality to an ordinary cigarette (prototype). It should be able to weaken the smoker's craving for smoking for a while or cause psychophysiological sensations, similar to smoking cigarettes. This is a broader class of tools than ANDS, since, in addition to ANDS, it also includes nicotine-free products, such as herbal cigarettes and nicotine-free e-cigarettes.

Comparison of RMSSD by stages of cigarette testing

Initially, based on the results of each study, we obtained four groups of data reflecting fluctuations in the RMSSD value at four successive stages of testing nicotine-free cigarettes, namely, control (I), imitation (II), the first cigarette (III) and the second cigarette (IV). Considering the duration of the tests and the range of impacts at each stage, to assess the effect of smoke on the ANS, it can be conventionally assumed that there is no direct relationship between stages I, II and III (that is, these are conditionally independent samples). In other words, each previous stage had a minimal impact on each subsequent one. Clearly, the same cannot be said about stages III and IV. In the context of our study, it was important to establish a statistically significant effect of smoke on ANS for any number of cigarettes smoked. In fact, this is what happens in real life: smokers alternate smoking cigarettes at intervals of 30 min or more, depending on the need and the ability to smoke.

At the first stage, we evaluated the RMSSD indicators in the groups as they are, without any preliminary manipulation.

Table S2 discloses the results of the multiple range tests for the RMSSD indicator with and without taking into account the smoking session.

Table S2. Multiple range tests (for the RMSSD indicator) with and without taking into account the smoking session.

	**(-) Smoking Session**			**Homogeneous Groups***		**(+) Smoking Session**			**Homogeneous Groups**	
	**Count**	**Mean**	**Median**	**1****	**2*****	Count	Mean	Median	1	2
I	4	17.45	17.1	X	X	4	17.45	17.1	X	X
II	4	20.53	20.4	X	X	5	20.84	21.3	X	X
II	5	20.3	20.0	X	X	6	21.22	20.6	X	X
IV	5	24.58	25.2	X	X	6	25.12	25.4	X	X

* Homogenous groups are identified using columns of X's. Within each column, the levels containing X's form a group of means within which there are no statistically significant differences. ** 1–Bonferroni procedure (overly conservative method) *** 2–Duncan`s method (overly liberal method).

The nonparametric Kruskal-Wallis test assessed intergroup differences as a whole as statistically significant at p = 0.00611 (excluding data in a smoking session) and p = 0.004423 (including data in a smoking session). The Kruskal-Wallis method tests the null hypothesis that the medians within each of the 4 columns are the same. Since the P-value is less than 0.05, there is a statistically significant difference among the medians at the 95.0% confidence level. The test also revealed statistically significant differences between the groups when compared in pairs (Table S3).

Table S3. Estimated difference between each pair of means (Bonferroni procedure).

**Contrast**	**Significance**	**Difference**	**+/- Limits**
II–II		0.225	2.67262
II–IV	*	−4.055	2.67262
III–IV	*	−4.28	2.51977

Absolutely identical results in paired comparisons were obtained regardless of the inclusion in the analysis of the values obtained directly at the time of the smoking session or its simulation. Indirectly, this suggests that the smoke did not cause psychophysiological stress in the subject directly at the time of smoking a nicotine-free cigarette. The analysis for the normality of samples also spoke about this. That is, regardless of the chosen statistical procedure for evaluating paired intergroup differences (conservative or liberal), based on the analysis of unadjusted RMSSD values, the two preliminary conclusions can be drawn. The first conclusion refers that in comparison with imitation smoking, inhalation of smoke from the first smoked cigarette did not have any effect on the HRV indicator during at least 90 min, and the second conclusion is that after smoking the second cigarette, the RMSSD indicator statistically significantly increased relatively to all previous stages of the test.

In view of the emerged trend in RR intervals, which became noticeable already in the first hour after the start of the test, it became necessary to correct the RMSSD values taking this factor into account. To determine pairwise differences between groups of data, the Game-Howell procedure, which is insensitive to the type of distribution and the size of samples, was used. This is due to the fact that after the correction of the RMSSD indicator by the value of RR intervals, problems arose with the normal distribution in group III (smoking the first cigarette). Although this procedure is nonparametric and is not particularly sensitive to the normal distribution. Nevertheless, one result obtained during the first smoking session had to be excluded from the analysis as an abnormal outlier. Thus, in further analysis, group III included 5 values of the RMSSD indicator. Taking into account the correction made, intergroup analysis of variance (ANOVA) clearly revealed

differences between groups at the p = 0.0014 level, and the Game-Howell test showed which pairs were statistically different in mean values (Table S4).

Table S4. Multiple Range Tests. Method: 95% Games-Howell.

	Count	Mean	Homogeneous Groups
I	4	18.9	XX
III	5	20.54	X
II	5	21.5	XX
IV	6	24.6333	X

Contrast	Significance	Difference	+/- Limits
II–IV	*	−3.13333	2.99879
III–IV	*	−4.09333	3.31429

* Statistically significant difference.

That is, even after the correction of the RMSSD indicator by the value of RR intervals, the statistically significant differences between the experimental and control groups remained the same. Hence, a third preliminary conclusion can be drawn, according to which one smoked cigarette may not be enough to obtain statistically reliable results of changes in parasympathetic activity, taking into account the HR trend. In further work with volunteers, we included in the trial consecutive smoking of two cigarettes with an interval of 1–1.5 h. The final test results for all volunteers are presented in Figures S1 and S2, as well as in Table S5.

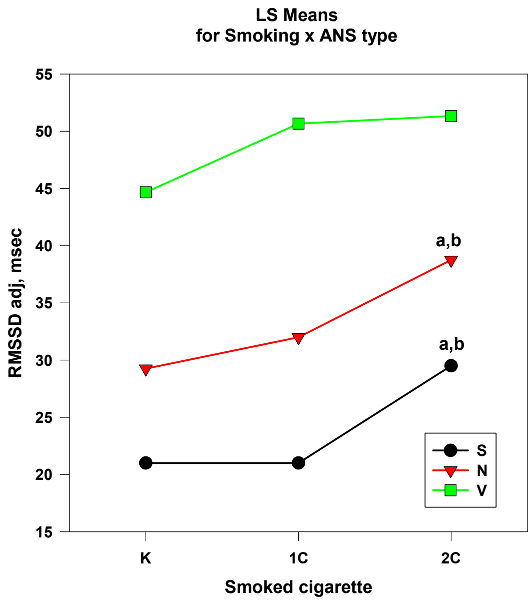

Figure S1. Dynamics of the average group adjusted RMSSD values in the process of testing nicotine-free cigarettes (1C stays for the first cigarette, while 2C refers to the second cigarette) relative to sham smoking (K), depending on the initial ANS type: S refers to sympathotonia (LF/HF > 2.5), N denotes normotonia (LF/HF = 0.7–2.0), while V designates vagotonia (LF/HF < 0.7), a refers to statistically significant difference with control

and b pertains to difference with the first cigarette.

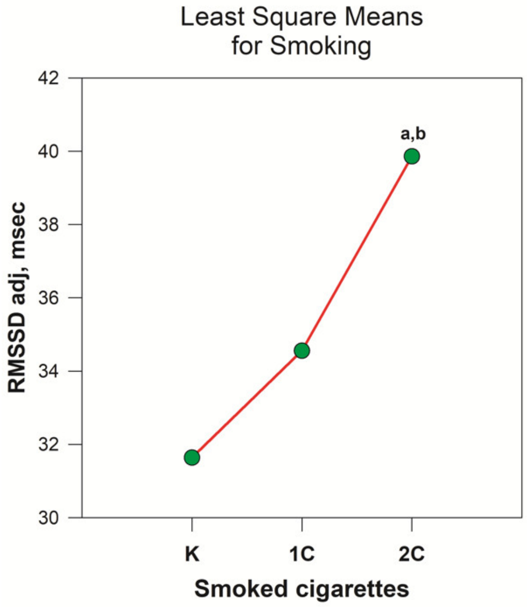

Figure S2. Dynamics of the group mean adjusted RMSSD values in the process of testing nicotine-free cigarettes (the same designations) relative to sham smoking (K) for all subjects.

Table S5. Two-way analysis of variance MANOVA of test results of a series of two nicotine-free cigarettes ("Smoking") in non-smokers with different initial vegetative status (ANS*).

General Linear Model					
Dependent Variable: RMSSD adj, msec					
**Normality Test:**	Passed	(P = 0.370)			
**Equal Variance Test:**	Passed	(P = 0.102)			
**Source of Variation**	**DF**	**SS**	**MS**	**F**	**P**
Smoking	2	375.339	187.669	12.777	<0.001
ANS type	2	3240.293	1620.146	110.308	<0.001
Smoking x ANS type	4	62.480	15.620	1.063	0.396
Residual	24	352.500	14.687		
Total	32	4057.515	126.797		

*The basic vegetative status of the subjects was determined on the basis of the ratio of LF/HF obtained from the spectral analysis of HRV. It was classified in three categories, namely, vagotonics (3 people), normotonics (4 people) and sympathotonics (4 people), depending on the dominance of the department of the ANS.

It clearly follows from the data in Table S5 that both the number of smoked cigarettes and the initial type of vegetative activity of the subjects influenced RMSSD. A more detailed analysis of multiple paired differences and the Holm-Sidak method from the SigmaPlot package showed that only in the vagotonics group there were no statistically significant differences between the experimental stages and control. In the remaining groups (volunteers with low and moderate initial levels of vagal activity), the differences acquired statistically significant outlines at the stage of repeated smoking.
